# Voluntarily wheel running protects doxorubicin-induced kidney injury by inhibiting oxidative stress through mitochondrial function

**DOI:** 10.1371/journal.pone.0321121

**Published:** 2025-04-01

**Authors:** Xin Jiang, Zeyu Wang, Longyun Wang, Yuqi Wang, Lijing Zhao, Hongyu Jiang

**Affiliations:** 1 Department of Rehabilitation, Physical examination center of the first Hospital of Jilin University, Changchun, Jilin, China; 2 Department of Rehabilitation, School of Nursing, Jilin University, Changchun, China; Suez Canal University, EGYPT

## Abstract

**Background:**

Doxorubicin (DOX) has a broad anticancer spectrum and precise anticancer effects, but its clinical application is limited by severe multiorgan toxicity, among which nephrotoxicity is one of the main adverse reactions. In this study, the protective effect of voluntary wheel running on nephrotoxicity induced by DOX was observed, and its mechanism was initially discussed.

**Methods:**

Forty male C57BL/6 mice were randomly divided into a control group (CTR), a voluntary wheel running group (EX), a doxorubicin model group (DOX) and a doxorubicin combined with voluntary wheel running group (COM). After 2 weeks of exercise, the mice were sacrificed. Serum creatinine (CREA), urea nitrogen (BUN), uric acid (UA), carbon dioxide combining power (CO_2_-CP), renal tissue apoptosis, oxidative stress and mitochondrial function indicators were assessed.

**Results:**

Compared with those in the DOX group, the concentrations of CREA, BUN and UA decreased, the number of TUNEL-positive cells in kidney tissue decreased, the expression of antiapoptotic proteins increased, and the expression of proapoptotic proteins decreased in the COM group. In addition, the COM can reduce the ROS and MDA contents in kidney tissue, reduce peroxide accumulation and alleviate mitochondrial respiratory chain damage caused by DOX.

**Conclusions:**

Voluntary wheel running can improve the mitochondrial function of renal cells and reduce oxidative stress damage, thus playing a protective role against nephrotoxicity caused by DOX. This study provides a new way to reduce the adverse reactions to chemotherapy in combination with the application of chemical drugs.

## Introduction

Anthracyclines, represented by doxorubicin (DOX), can inhibit the growth of several solid and hematologic malignant tumors and are currently among the most effective antitumor drugs [1]. However, its severe adverse effects, such as nephrotoxicity and cardiotoxicity, limit its clinical application [2]. Doxorubicin-induced kidney injury not only affects patients’ renal function but may also lead to acute or chronic kidney disease, thereby impacting patients’ long-term prognosis and quality of life [3]. To reduce nephrotoxicity caused by DOX, nephrologists and oncologists have made various efforts [4], such as trying nanomedicine delivery systems (NDDSs) [1,5]; however, the specific mechanism of nephrotoxicity is unclear, and the effect is not ideal. Therefore, clarifying the specific mechanism of damage caused by DOX is highly important for the treatment of adverse reactions and its clinical application.

The detailed underlying mechanism of DOX-induced nephrotoxicity remains unknown. DOX can cause renal damage by enhancing oxidative stress and producing excessive reactive oxygen species [6–11] while reducing the activity of the antioxidant enzymes catalase (CAT) and superoxide dismutase (SOD) [12], leading to swelling and necrosis of renal tubular epithelial cells and ultimately causing renal dysfunction. Other studies have shown that DOX can also affect the normal function of mitochondria and play an apoptotic role by disrupting mitochondrial pathways [8,13,14], further affecting energy metabolism. Therefore, improving mitochondrial function and reducing ROS production may be effective strategies for alleviating nephrotoxicity caused by DOX. In recent years, nonpharmacological interventions have garnered widespread attention for their potential in mitigating chemotherapy-induced nephrotoxicity. For example, exercise interventions have attracted interest because of their potential to reduce kidney damage by improving systemic blood flow, modulating inflammatory responses, and enhancing antioxidant capacity [15]. These studies not only expand the approaches for managing chemotherapy-induced nephrotoxicity but also offer potential safe and drug-free treatment options.

Aerobic exercise, a low-cost nonpharmacological intervention, has been shown to serve as a potential protective strategy to mitigate doxorubicin-induced cardiac damage [16]. Similar to cardioprotective exercise, aerobic exercise has been reported to improve renal apoptosis by influencing endogenous and exogenous apoptotic pathways in a rat model after a single injection of DOX [17–19]. In addition, 60 minutes of treadmill training was able to reduce glomerular swelling and renal interstitial and cortical collagen deposition [20]. Mariana, Y., et al. noted that exercise alleviates kidney damage by modulating the expression of antioxidants and inflammatory factors, thereby providing an effective protective strategy for chemotherapy patients [21]. Boeno et al. reported that exercise preconditioning can prevent tissue toxicity caused by DOX by increasing antioxidant capacity, HSP70, and SIRT1/3 [22]. Ana et al. reported that patients engaging in aerobic exercise showed marked improvement in kidney function during chemotherapy, with lower indicators of kidney damage. The study recommends that regular aerobic exercise be included as an adjunct therapy for chemotherapy patients to protect the kidneys and enhance quality of life [23]. These findings suggest that aerobic exercise has a favorable protective effect on DOX-induced nephropathy. On the basis of the favorable results of exercise after a single dose of DOX, it is expected that repeated dosing will better benefit the maintenance of renal structure to more closely mimic clinical treatment [24]. Although existing studies have shown that exercise can improve mitochondrial biogenesis and function and reduce oxidative stress, its specific mechanisms in chemotherapy-related kidney injury still need further clarification [25]. This study explores the protective effects of voluntary wheel running on doxorubicin-induced kidney injury, providing new insights into the molecular mechanisms of exercise in organ protection.

Therefore, this study used multiple injections of DOX-damaged mice as a model to investigate the protective effect of free roller motion on DOX-induced nephrotoxicity and its effects on oxidative stress and mitochondrial function, laying a foundation for the primary treatment of DOX injury via aerobic exercise. Furthermore, this study holds significant translational medical importance. Developing non-pharmacological interventions, such as exercise, to prevent or mitigate chemotherapy-induced organ injury can not only improve the quality of life for chemotherapy patients but may also provide a safe and cost-effective adjuvant treatment strategy for clinical practice.

## Materials and methods

### Reagents and antibodies

Doxorubicin hydrochloride (Yuanye Biotechnology Co., Ltd.) was purchased from Shanghai, China. Anti-B-cell lymphoma-2 (Bcl-2), anti-B-cell lymphoma-extra-large (bcl-xl), anti-Bcl2-associated X protein (Bax), anti-kelch-like ECH-associated protein-1 (Keap-1), anti-heme oxygenase-1 (HO-1), and anti-goat anti-rabbit IgG HRP antibodies were purchased from Affinity Biosciences (Cincinnati, USA). Anti-caspase3 (Cas3), anti-caspase9 (Cas9), anti-nicotinamide adenine phosphate quinone dehydrogenase 1 (NQO-1), anti-nuclear Factor E2-related factors (Nrf-2), anti-subcomplex of the stator of bovine mitochondrial ATP synthase (ATP5f1), anti-cytochrome C-1 (CYC1), anti-nicotinamide adenine dinucleotide dehydrogenase (ubiquinone) flavoprotein 1 (NDUFV1), anti-cytochrome C oxidase subunit 4 (COX4), anti-β-actin antibodies were purchased from Proteintech Group, Inc. (Chicago, USA).

### Animals

**Fig 1:**
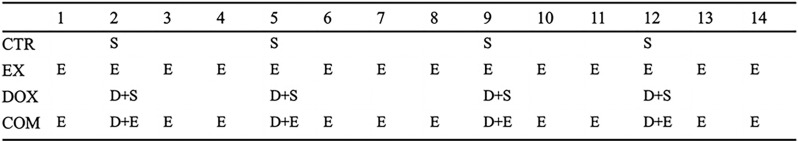
The timeline of animal experiments. D: DOX; E: voluntarily wheel running; S:Saline.

A total of 40 6-week-old C57BL/6 mice weighing 20 g were collected from Beijing Spaf Biotechnology Co., Ltd. The feeding and handling of experimental animals followed the management regulations of experimental animals of Jilin University, and the experimental protocol was approved by the Experimental Animal Ethics Committee of Jilin University (Changchun). Before the experiment began, we screened all the mice to ensure that they were able to complete the wheel running exercise within 1 hour. After one week of adaptation, each mouse was numbered, and a random number generator was used to assign them to their respective groups, ensuring an equal sample size in each group. The mice were randomly divided into four groups (n = 10): the control group (CTR), exercise group (EX), doxorubicin group (DOX) and exercise combined with doxorubicin group (COM). The sample size of 10 mice per group was based on empirical data and the design of previous similar experiments [22,26,27]. As shown in Fig 1, voluntary wheel running lasted for 14 days in the EX and COM groups; doxorubicin (2.5 mg/kg) was intraperitoneally injected into the DOX and COM groups on Days 2, 5, 9, and 12; and the other groups were intraperitoneally injected with the same volume of normal saline. The different groups of mice were subjected to the same amount of daily exercise, with 1,800 cycles of free roller exercise per day. After two weeks of exercise, the mice were subsequently sacrificed via pentobarbital overdose on Day 15, and serum and kidney samples were obtained. To minimize the suffering of the animals during the experiment, we regularly monitored the health of the experimental animals. Before and after the experiment, we strictly abided by the relevant regulations of animal ethics and took measures to ensure the comfort and health of the animals.

### H&E staining

The kidney samples were fixed with 4% paraformaldehyde and embedded in paraffin. The stones were cut into 4-μm-thick slices and fixed on slides. The slides were subsequently stained with hematoxylin and eosin (H&E) and observed under a light microscope at different magnifications (Olympus IX71, Olympus Corporation, Tokyo, Japan).

### Serum biochemical examination

On Day 15, the serum was collected and centrifuged at 3000×g at 4°C for 15 minutes. The creatinine, urea nitrogen, uric acid, and carbon dioxide binding rates were obtained from Jiancheng Biotechnology (Jiangsu, China). All operations were performed in accordance with the manufacturer’s instructions. Creatinine (CREA) (Item No. C011-2-1), blood urea nitrogen (BUN) (Item No. C013-2-1), uric acid (UA) (Item No. C012-2-1), and carbohydrate combining power (CO2-CP) (Item No. C028-1-1).

### TUNEL assay

The terminal deoxynucleotidyl transferase-mediated dUTP-biotin nick end labeling (TUNEL) assay was employed to analyze apoptosis. Renal cell apoptosis was observed via fluorescence microscopy. Images were acquired with an Olympus IX71 digital camera (Tokyo, Japan). The apoptotic index (positive cells/total cells ×  100) was calculated for each section.

### Measurement of ROS

The ROS fluorescent probes were used according to the manufacturer’s protocol (KGAF019). The kidney tissue was ground with cold PBS at 4°C and centrifuged at 100 ×  g for 3‒5 min to collect the supernatant, and 2 µl of DHE was added every 100 µl to the 96-well plate and incubated for 90 min in the dark. Finally, the cells were imaged via fluorescence microscopy (Optitec-YG-100). The fluorescence intensity was quantified via ImageJ.

### The levels of oxidative stress markers

On Day 15, kidney tissue was collected and frozen in 4% paraformaldehyde or −80°C. The concentrations of malondialdehyde (MDA) (Item No. S0131M), superoxide dismutase (SOD) (Item No. S0109), glutathione (GSH) (Item No. S0053), glutathione oxide (GSSG) (Item No. S0053) and peroxidase (CAT) (Item No. S0051) were measured in 10% kidney tissue homogenate.

All procedures were carried out according to the manufacturer’s requirements (Beyotime, Shanghai, China).

### Mitochondrial complexes and ATP content analysis

A mitochondrial isolation kit was used to extract mitochondria from kidney tissue (Beyotime, Shanghai, China). The purified mitochondria were assayed for mitochondrial complex I, II, III, IV and V activity via the Mitochondrial Complex Activity Assay Kit (Jiancheng, Nanjing, China), and adenosine triphosphate (ATP) was evaluated according to the kit instructions (Beyotime, Shanghai, China).

### Western blot assays

Total or mitochondrial protein samples were extracted from 40 mg of kidney tissue with 400 µl of cold RIPA containing protease inhibitors. The proteins were separated by 12.5% SDS‒PAGE and then transferred to polyvinylidene fluoride (PVDF) membranes (Immobilon-P, Millipore, Merck, Italy) in SDS‒PAGE transfer buffer (Servicebio, China). The PVDF membrane was incubated in 5% skim milk for 1 h, and the membrane was incubated with diluted primary antibody at 4°C overnight after being washed with TBST. The membrane was washed with TBST again and incubated with a secondary antibody labeled with horseradish peroxidase for 1 hour. Finally, enhanced chemiluminescence (ECL) was used to detect protein levels. The strip is quantified via ImageJ. GAPDH and β-tubulin were used as internal parameters for total protein, and COX4 was used as an internal parameter for mitochondrial protein. The Protein marker is Blue Plus® V Protein Marker (10–190 kDa) (Item: DM141-01).

### Statistical analysis

In both the experimental process and data analysis stages, all personnel conducting the experiments or analyses were blinded to the sample grouping. Sample numbers were randomly assigned and managed by independent technical staff, ensuring that experimenters or analysts operated solely on the basis of sample numbers without access to grouping information, thereby maintaining the independence of experimental operations. The values are expressed as the means ±  standard deviations. The data were analyzed via SPSS 22.0 and GraphPad Prism version 8.0. One-way ANOVA followed by Tukey’s multiple comparisons test was performed to evaluate differences between the two groups, and *P* < 0.05 was considered statistically significant.

## Results

### Voluntary wheel running alleviates renal damage induced by DOX

**Fig 2:**
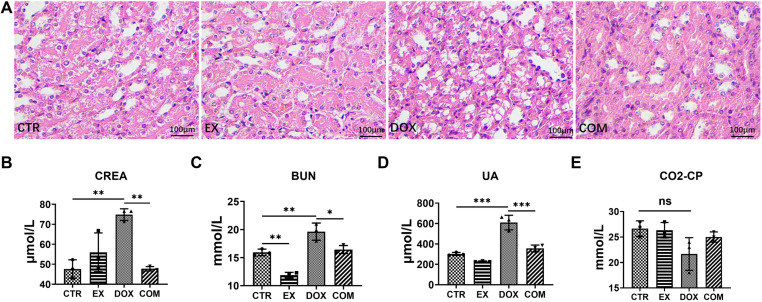
Effect of voluntarily wheel running on DOX-induced renal dysfunction. (A) H&E staining of kidney tissue section (×200). (B–E) The concentration of CREA, BUN, UA, CO2-CP in serum. * P < 0.05, **P < 0.01 and ***P < 0.001.

To understand whether voluntary wheel running can alleviate renal damage caused by DOX, renal tissue morphology was observed via H&E staining, and the serum levels of CREA, BUN, UA, and CO_2_-CP were detected. As shown in Fig 2A, the glomerular structure of the CTR group was normal, and the tubular cells were arranged neatly, without vacuoles, deformation or necrosis. In the EX-group, a small number of renal tubular epithelial cells were disordered and formed vacuoles. The renal tubule interstitial region was enlarged in the DOX group, the epithelial cell cytoplasm was significantly vacuolated, and the cells were swollen and deformed, whereas the renal tubule epithelial cells were slightly vacuolated in the COM group. As shown in Fig 2B-E, CREA, BUN, and UA levels were significantly greater in the DOX group than in the control group (95% CI = −43.07 ~ −11.34, *p* =  0.0020; 95% CI = −6.333 ~ −1.070, *p* =  0.0072; 95% CI = −421.6 ~ −193.1, *p* <  0.001). Moreover, com*p*ared with those in the DOX group, the levels of CREA, BUN and UA in the COM group were significantly lower (95% CI = 11.23 ~ 42.95, *p* =  0.0021; 95% CI = 0.560 ~ 5.823, *p* =  0.0175; 95% CI = 138.7 ~ 367.2, *p* < 0.001). However, there were no significant differences in CO2‒CP levels among the grou*p*s. These data indicate that voluntary wheel running alleviates the renal damage caused by DOX.

### Voluntary wheel running alleviates renal cell apoptosis induced by DOX

**Fig 3:**
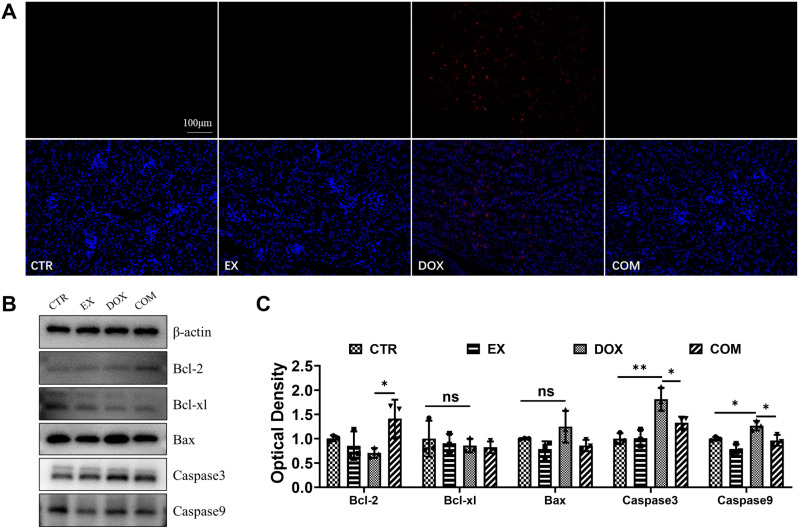
Effect of voluntarily wheel running on DOX-induced apoptosis of renal cells. (A) TUNEL staining of renal tissue sections ( × 200). (B) Apoptosis-related proteins Bcl-2, bcl-xl, Bax, Caspase3, Caspase9 levels in renal tissue. (C) Western blots quantitative analysis. * *P* <  0.05, **P < 0.01 and ***P < 0.001.

To understand whether voluntary wheel running could alleviate the apoptosis induced by DOX, renal cell apoptosis was detected via TUNEL staining, and the protein expression of Bcl2, Bcl-xl, Bax, Caspase3 and Caspase9 was detected via Western blotting. As shown in Fig 3A, red fluorescence indicated apoptosis, and only red fluorescence was detected in the DOX group. Western blotting was used to further detect apoptosis-related protein expression, and β-actin protein was used as a loading control, as shown in Fig 3B and C. Compared with those in the CTR group, the expression levels of the proapoptotic proteins Caspase3 and Caspase9 were significantly upregulated in the DOX group (95% CI = −1.296 ~ −0.3322, *P* = 0.0022; 95% CI = −0.5283 ~ −0.010, *P* = 0.0410). Compared with those in the DOX group, the expression of bcl2 was upregulated (95% CI = −1.420 ~ −0.001, *P* = 0.0496), while the expression of Caspase3 and Caspase9 were downregulated in the COM group (95% CI = 0.003 ~ 0.9678, *P* =  0.048; 95% CI = 0.043 ~ 0.5612, *P* =  0.0219). These results show that voluntary wheel running can alleviate renal cell apoptosis induced by DOX.

### Voluntary wheel running alleviates oxidative stress induced by DOX

**Fig 4:**
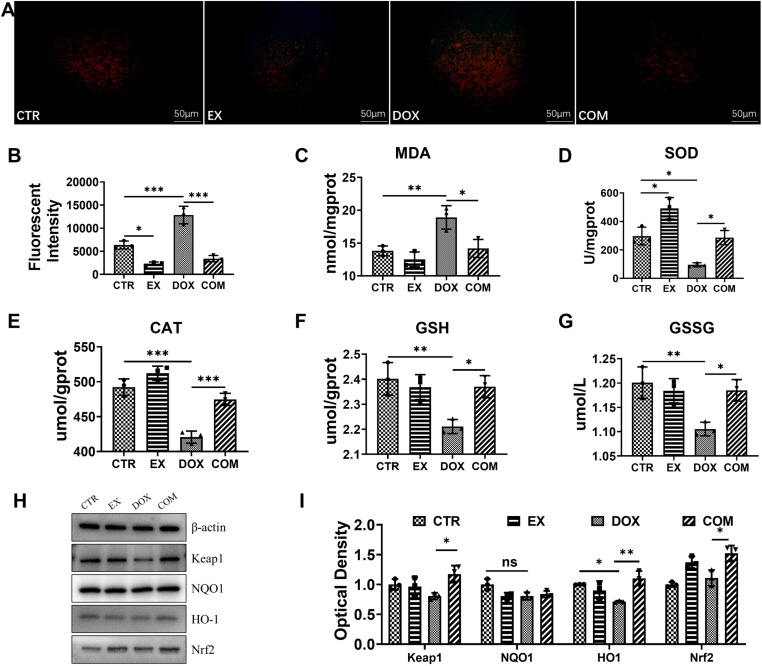
Effect of voluntarily wheel running on DOX-induced oxidative stress. (A) ROS fluorescence staining intensity (×200). (B) Quantitative analysis of ROS levels in renal tissue. (C-G) MDA, SOD, CAT, GSH, GSSG activity in renal tissue. (H) Expression levels of Keap-1, NQO-1, HO-1, Nrf-2 in renal tissue. (I) Western blot quantitative analysis. * P < 0.05, **P < 0.01 and ***P < 0.001.

To further understand the protective mechanism of voluntary wheel running on renal cells, we examined the oxidative stress level in renal tissue. As shown in Fig 4A, red fluorescence represents the level of ROS. Compared with that in the CTR group, the fluorescence intensity in the DOX group was significantly greater (*P* < 0001), and compared with that in the DOX group, the fluorescence intensity in the EX and COM groups was lower (*P* < 0.05). The results of the quantitative analysis revealed that the ROS level in the DOX group was the highest (Fig 4B). The biochemical indices of oxidative stress-related markers in kidney tissue were also examined. As shown in Fig C–G, the MDA content was significantly greater in the DOX group than in the CTR group (95% CI = −8.833 ~ −1.356, *P* = 0.0088), and the SOD (95% CI = 44.22 ~ 359.2, *p* = 0.0127), CAT (95% CI = 43.59 ~ 99.80, *p* < 0.001), GSH (95% CI = 0.05256 ~ 0.3285, *p* = 0.0081), and GSSG (95% CI = 0.02628 ~ 0.1643, *p* = 0.0081) activities were lower. Compared with those in the DOX group, the MDA content was lower in the COM group (95% CI = 0.9846 ~ 8.462, *p* = 0.0138), while the SOD (95% CI = −348.3 ~ −33.27, *p* = 0.0176), CAT (95% CI = −82.14 ~ −25.93, *p* < 0.001), GSH (95% CI = −0.2970 ~ −0.02104, *p* = 0.0234) and GSSG (95% CI = −0.1485 ~ −0.01052, *p* = 0.0234) activities were significantly greater. To explore the expression of oxidative stress-related proteins, Western blot analysis was performed, and β-actin antibodies were used as loading controls. As shown in Fig H and I, HO1 expression was significantly lower in the DOX group than in the CTR group (95% CI = 0.0008 ~ 0.5662, *P* =  0.0430), and compared with that in the DOX group, the expression of keap1, HO1 and Nrf2 in the COM group was significantly greater (95% CI = −0.7026 ~ −0.03491, *P* = 0.0296; 95% CI = −0.6702 ~ −0.1124, *P* =  0.0073; 95% CI = −0.7157 ~ −0.1068, *P* =  0.0093). However, there was no significant difference in the expression of NQO1. These results show that voluntary wheel running can alleviate the oxidative stress induced by DOX.

### Voluntary wheel running alleviates mitochondrial dysfunction induced by DOX

**Fig 5:**
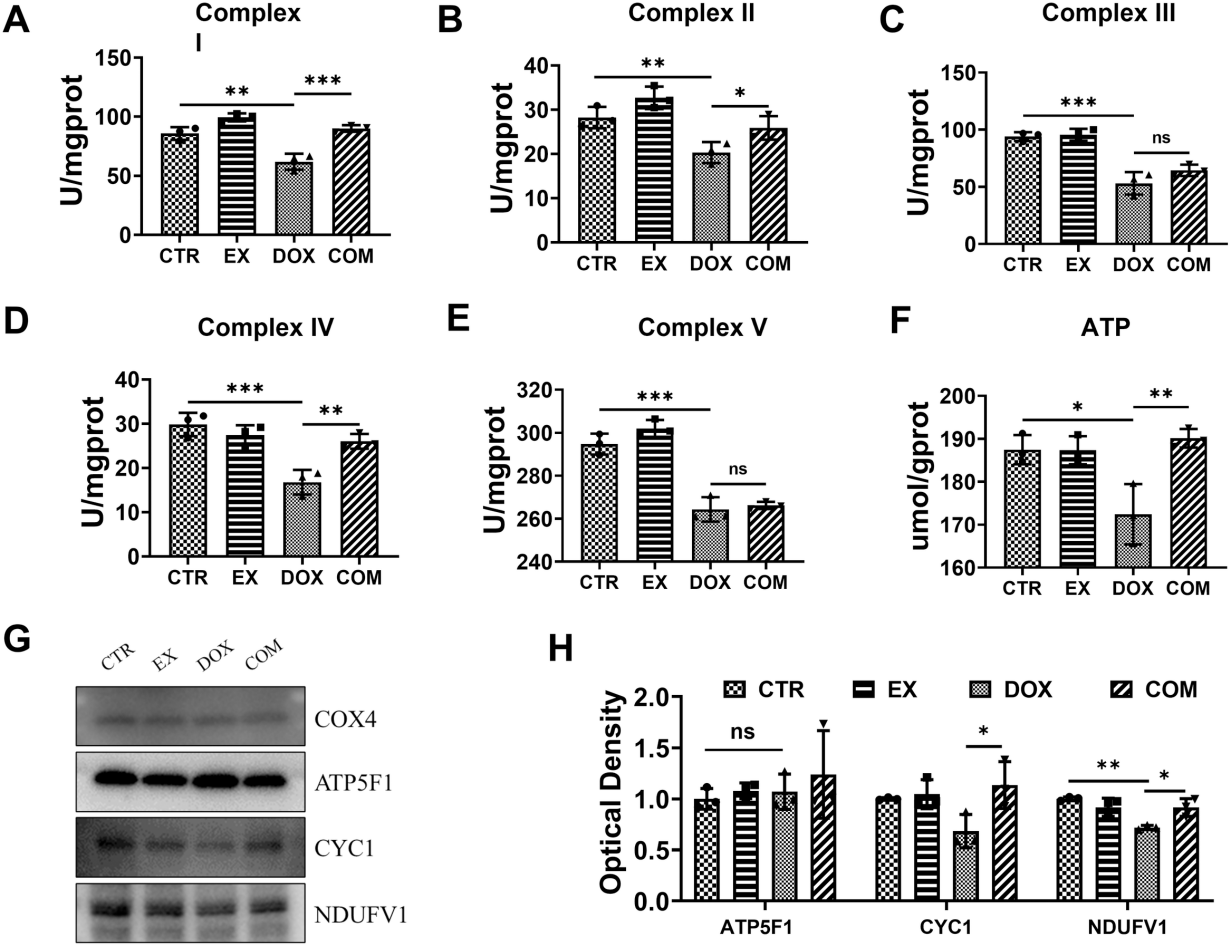
Effect of voluntarily wheel running on DOX-induced mitochondrial dysfunction. (A–E) Levels of mitochondrial complex I-V. (F) ATP content in renal tissue. (G) Expression levels of ATP5F1, CYC, NDUFV1 in mitochondria. (H) Western blot quantitative analysis. * P < 0.05, **P < 0.01 and ***P < 0.001.

ROS play a key role in oxidative stress, and electron transport errors in the mitochondrial respiratory chain cause the accumulation of ROS [28]. To understand whether exercise has a protective effect on DOX-induced mitochondrial dysfunction, this study first detected mitochondrial respiratory chain complex enzyme activity in kidney tissue and used COX4 as a loading control to detect the expression of mitochondrial respiratory chain-related proteins. As shown in Fig 5A–E, compared with those in the CTR group, the activities of mitochondrial complexes I, II, III, IV, and V in the DOX group were significantly lower (95% CI = 10.05 ~ 37.76, *P* = 0.0019; 95% CI = 2.219 ~ 14.78, *P* = 0.0092; 95% CI = 22.76 ~ 59.11, *P* < 0.001; 95% CI = 6.295 ~ 19.76, *P* < 0.001; 95% CI = 17.98 ~ 42.67, *P* < 0.001). Compared with that in the DOX group, the activity of mitochondrial complexes I, II, and IV was greater in the COM group (95% CI = −41.99 ~ −14.28, *P* < 0.001; 95% CI = −12.57 ~ −0.006724, *P* = 0.0497; 95% CI = −15.96 ~ −2.495, *P* = 0.0085), but the activity of mitochondrial complexes III and V was not significantly different. The ATP content in each group was significantly greater in the CTR and COM groups than in the DOX group (95% CI = 2.606 ~ 27.47, *P* = 0.0178; 95% CI = −30.13 ~ −5.269, *P* = 0.0067) (Fig 5F). To further investigate the effects of voluntary wheel running on mitochondrial function, Western blot analysis was performed to detect the expression of the mitochondrial complex enzyme subunit markers NDUFV1, CYC-1 and ATP5f1. As shown in Fig 5G and H, the results revealed that the CYC1 and NDUFV1 contents in the COM group were greater than those in the DOX group (95% CI = −0.9002 ~ −0.0007, *P* = 0.0496; 95% CI = −0.3756 ~ −0.01466, *P* = 0.0332). These results show that voluntary wheel running can alleviate the mitochondrial dysfunction induced by DOX.

## Discussion

DOX is a broad-spectrum antineoplastic drug. However, its multiple organ toxicity limits its clinical application [22,29,30]. The kidney is the organ that metabolizes and clears DOX, so it is prone to functional impairment caused by DOX [18,31]. To date, there is no effective intervention method for treating renal tissue damage caused by DOX. An increasing number of studies have shown that oxidative stress and apoptosis are the main causes of DOX nephrotoxicity [32]. Therefore, finding a safe and effective strategy to reduce the content of oxygen free radicals in the kidney and inhibit cell apoptosis to protect kidney cells from DOX-induced cytotoxicity is meaningful and necessary.

Studies have shown that antioxidants such as apigenin [4] have a protective effect against DOX-induced nephrotoxicity, primarily by reducing oxidative stress and inflammatory responses. These antioxidants are characterized by direct targeting of free radicals and inflammation. In contrast to pharmacological interventions such as dexrazoxane [18,19], the protective effect of voluntary exercise does not rely on exogenous compounds, but is achieved by activating endogenous antioxidant networks such as Nrf2 and SOD, thereby avoiding potential toxic risks. In addition, although natural antioxidants such as curcumin have shown efficacy in animal models [30], their clinical use is limited by low bioavailability, which can be circumvented by exercise interventions. Voluntary wheel running, as a nonpharmacological intervention, offers a systemic approach rather than being limited to specific molecular targets, which may provide a greater advantage in the overall regulation of the antioxidant enzyme system.

Exercise may confer protection by activating the Nrf2 pathway [33], thereby increasing the expression of antioxidant enzymes such as SOD, CAT, and HO-1, which play crucial roles in scavenging free radicals and reducing cellular damage. Additionally, exercise may alleviate inflammatory responses by inhibiting the NF-κB pathway [34]. The NF-κB pathway is pivotal in cellular stress and inflammatory responses, and exercise can suppress its activation, thereby reducing inflammation. Furthermore, exercise may improve mitochondrial respiratory chain function, reduce ROS accumulation and consequently mitigate oxidative stress and apoptosis [35]. Toyama et al. demonstrated that exercise therapy may be an effective clinical strategy for improving kidney function [36], but the mechanism of action is not clear. This study verified that voluntary wheel running can reduce DOX-induced renal cell injury to some extent and explored its mechanism.

First, this study explored whether voluntary wheel running can reduce DOX-induced nephrotoxicity [5,37,38]. DOX can induce renal dysfunction in mice, which manifests as swelling and necrosis of renal tubular epithelial cells, severe vacuolation of interstitial cells, and increased serum CREA, BUN, and UA levels. After voluntary wheel running for two weeks, the symptoms of renal insufficiency in the mice were alleviated. Consistent with the findings of Moecke et al. [39], aerobic exercise can alleviate kidney injury by reducing the area of kidney necrosis.

Apoptosis is one of the main causes of nephrotoxicity. Kwak et al. reported that exercise attenuates the age-induced increase in the Bax/Bcl-2 ratio and increases Bcl-2 levels in the rat heart by decreasing Bax protein expression, thereby reducing caspase-9 levels, suggesting that endurance exercise training has a protective effect on apoptosis [40]. Consistent with these findings, this study revealed that DOX upregulated the protein expression of Bax, caspase3 and caspase9, and these phenomena were reversed after voluntary wheel running. In addition, TUNEL staining more directly demonstrated that free roller exercise alleviated renal cell apoptosis. Therefore, voluntary wheel running can reduce kidney injury by increasing DOX-induced apoptosis.

Unbalanced oxidative stress leads to cell cycle arrest and cell apoptosis. Oxidative stress is triggered by the overproduction of ROS, which has traditionally been thought of as an imbalance between pro-oxidant and antioxidant homeostasis [41]. In the case of low levels of free radicals, intracellular antioxidants such as SOD, CAT, and glutathione peroxide (GSH-Px) can reduce the degree of cellular damage caused by oxidative stress [42,43]. De Souza et al. [44] performed 5/6 renal removal in rats and then treadmill training and reported that physical exercise can improve antioxidant defense systems such as SOD and GPX, thus reducing superoxide production and renal tissue oxidative damage. In this study, compared with the control, DOX led to a significant decrease in SOD activity and GSH levels, accompanied by an increase in MDA, resulting in the overproduction of ROS and the induction of oxidative damage. In addition, voluntary wheel exercise reduced the MDA content in kidney tissue and increased the activities of the antioxidant substances SOD, CAT and GSH, thereby reducing the accumulation of lipid peroxides.

We further investigated the role of antioxidant signaling in the effectiveness of voluntary wheel running. Mitochondria are the center of cellular energy metabolism, and normal mitochondrial function is crucial for maintaining cell health. Mitochondria not only generate ATP but also play a role in regulating the intracellular redox state. When mitochondrial function is impaired, excess ROS are produced [35,45], leading to oxidative stress. Oxidative stress can damage cellular structures, affect cellular functions, and trigger apoptosis. Excess ROS can activate apoptotic pathways, guiding cells toward programmed cell death. This process plays a significant role in the progression of various diseases, such as cancer and neurodegenerative disorders. Nrf2 is a transcription factor responsible for regulating the antioxidant stress response. When cells sense oxidative stress, Nrf2 is translocated to the nucleus, where it activates the expression of a series of antioxidant genes, thereby increasing the antioxidant capacity of the cell. Research has shown that activation of Nrf2 can protect cells from oxidative damage by inhibiting ROS produced by mitochondria, thereby delaying apoptosis [46].

Activated Nrf2 is transported to the nucleus and binds to the transcriptional regulatory region of several antioxidant agents, such as HO1 and NQO1, to promote its expression [47,48]. Keap1 can negatively regulate the polymerization or dissociation of Nrf2, thereby controlling Nrf2 to regulate the expression of various antioxidant enzymes to combat oxidative stress [49,50]. The results of this study revealed that keap1/NQO1/HO-1/Nrf-2 signaling pathway activation was greater in the COM group than in the DOX group. Similarly, the review of Mallard et al. [33] also reported that exercise can increase the protein expression of Nrf2. This study showed that voluntary wheel running significantly activated the renal Nrf2 pathway, which is consistent with Tebay et al. [51], that exercise reduces oxidative damage through an Nrf2-dependent antioxidant defense system. In addition, exercise may maintain cellular energy homeostasis by selectively clearing DOX-induced dysfunctional mitochondria via PINK1/ Parkin-mediated mitochondrial autophagy pathway [52].Therefore, voluntary wheel running activates the antioxidant pathway, resulting in rapid clearance of ROS.

Mitochondria, as the primary sites of energy metabolism, produce ATP through OXPHOS and are involved in many other metabolic pathways [47]. Moreover, the release of the mitochondrial complex enzyme subunit labeled with cytochrome c can trigger the activation of caspase-9, and studies have shown that exercise training effectively inhibits this activation [17]. Therefore, in this study, mitochondrial respiratory chain complex enzyme activity and mitochondrial complex enzyme subunit markers were detected to determine whether mitochondrial function was impaired. Consistent with previous studies, DOX can induce mitochondrial dysfunction, especially in the electron transport system, which is dependent on ATP synthesis, and voluntary wheel exercise can increase the activity of mitochondrial complexes I, II, and IV, upregulate complex-related subunit expression, and increase ATP levels. The above results show that voluntary wheel exercise can reduce the mitochondrial damage induced by DOX and prevent the release of cytochrome c in mitochondria, thereby reducing the degree of renal cell apoptosis.

Mice are commonly used models for studying the toxicity of chemical drugs such as DOX, and investigating the mechanisms of nephrotoxicity in mice can help us understand similar mechanisms in humans. The similarities between humans and mice in terms of oxidative stress and inflammatory responses, particularly in the regulation of pathways such as Nrf2 and NF-κB, suggest that findings from mouse experiments may be relevant to humans. Exercise interventions in mouse models provide preliminary support for clinical applications, potentially serving as a nonpharmacological approach to aid in the prevention and mitigation of nephrotoxicity in chemotherapy patients.

Limitations: ① This study only used male mice, which may affect the generalizability of the results. Studies have shown sex-based differences [53,54] in response to DOX treatment and exercise interventions [55], potentially due to hormonal and metabolic variations. These factors may influence oxidative stress levels, inflammatory responses, or therapeutic outcomes, which could result in different effects in female subjects. Future studies should include both male and female mice to fully explore potential sex differences in response to DOX and exercise interventions, providing a more comprehensive understanding of these interactions across sexes. ② This study is based solely on an animal model, and further preclinical research is needed to confirm the mechanisms and effects of exercise in protecting against DOX-induced nephrotoxicity, providing a stronger foundation for potential future clinical applications. ③ The exercise intervention in this study was relatively short, and more long-term research on the effects of exercise on chemotherapy outcomes should be conducted in the future.

**Fig 6:**
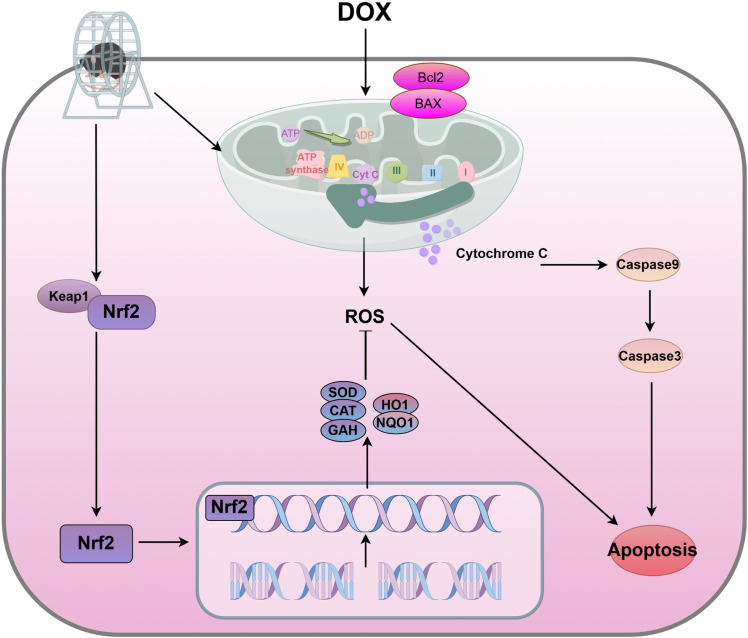
Illustration of voluntarily wheel running alleviating renal toxicity induced by DOX. (Abbreviations: I, complex I; II, complex II; III, complex III; IV, complex IV; V, complex V) (by figdraw).

In summary, this study suggests that voluntarily wheel running can protect against DOX-induced kidney injury by improving mitochondrial function and inhibiting oxidative stress (Fig 6).

## Supporting information

S1 File Detailed description of the biochemical assay methods for oxidative stress. (DOCX)

S2 File Western blot staining of whole membrane. (DOCX)
