# Supplementary material for: Voluntarily wheel running protects doxorubicin-induced kidney injury by inhibiting oxidative stress through mitochondrial function
Source: PLoS One. 2025 Apr 1;20(4):e0321121. doi: 10.1371/journal.pone.0321121 (PMC11960966; doi:10.1371/journal.pone.0321121)
Supplement: S1 File — (DOCX) [file pone.0321121.s001.docx]

**Supporting information 1:** Detailed description of the biochemical assay methods for oxidative stress.

**CAT Assay Method:** Based on a colorimetric reaction, catalase decomposes hydrogen peroxide into water and oxygen. The remaining hydrogen peroxide is catalyzed by peroxidase to oxidize a chromogenic substrate, producing a red product (maximum absorbance at 520 nm). Sensitivity: Detection of catalase activity was achieved at concentrations as low as 1 U/ml. Specificity: This characteristic is minimally affected by interference from other absorption peaks and is suitable for whole blood, red blood cell lysate, serum, and tissue homogenates. Validation Steps: A standard curve was prepared using hydrogen peroxide standards, and catalase activity was calculated in samples based on the curve. An equal volume of double-distilled water was used as a blank control, and the blank absorbance was subtracted to eliminate interference from nonspecific absorption (S0051; Beyotime).

**GSH and GSSG assay Method:** GSSG is reduced to GSH by glutathione reductase, and GSH reacts with the chromogenic substrate DTNB to produce a yellow product (TNB), which is detectable at 412 nm. Sensitivity: The assay’s detection limit is as low as 0.5 μM. Specificity: This method is designed specifically for detecting reduced and oxidized glutathione in biological samples and includes a protein removal step to ensure accurate detection without interference from proteins. Validation steps: GSSG standards were diluted to create a standard curve for determining the total and oxidized glutathione contents. The protein removal reagent M was used to ensure that the proteins did not interfere with the glutathione measurements, thus improving accuracy. The blank and total glutathione controls were used for the GSH and GSSG measurements to minimize error (S0053; Beyotime).

**SOD Assay Method:** In the NBT colorimetric method, superoxide anions generated by xanthine oxidase reduce NBT to form a blue formazan product. SOD reduces superoxide anions, thereby inhibiting formazan formation, with the absorbance measured at 560 nm. Sensitivity: This method is unaffected by endogenous hydrogen peroxide in samples. Specificity: The method effectively eliminates interference from antioxidants, providing high specificity in measuring SOD activity. Validation steps: Multiple blank controls (including controls with and without antioxidants) were used to ensure that there was no interference from sample components (S0109; Beyotime).

**The MDA assay method** is based on the reaction between MDA and TBA to form a red adduct, which can be quantified by colorimetry (maximum absorbance at 535 nm). Sensitivity: MDA concentrations as low as 1 μM and up to 200 μM were detected. Specificity: Includes antioxidants to prevent new MDA formation during the assay and breaks down some natural MDA polymers, improving measurement accuracy. Validation steps: MDA standards were diluted to different concentrations, and a standard curve was created to ensure that the sample measurements aligned with known concentrations. A blank control was used in all assay systems to ensure that absorption without samples did not affect the results (S0131; Beyotime).
