# Supplementary material for: Voluntarily wheel running protects doxorubicin-induced kidney injury by inhibiting oxidative stress through mitochondrial function
Source: PLoS One. 2025 Apr 1;20(4):e0321121. doi: 10.1371/journal.pone.0321121 (PMC11960966; doi:10.1371/journal.pone.0321121)

**Supporting information 2:** Western blot staining of whole membrane.

**1-Bcl2**

**
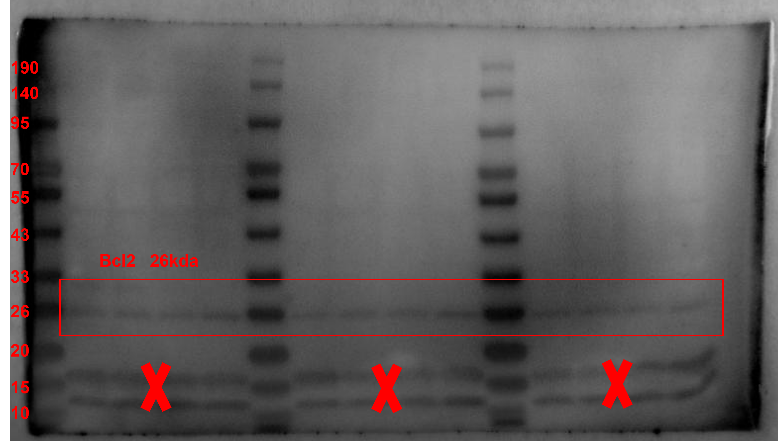

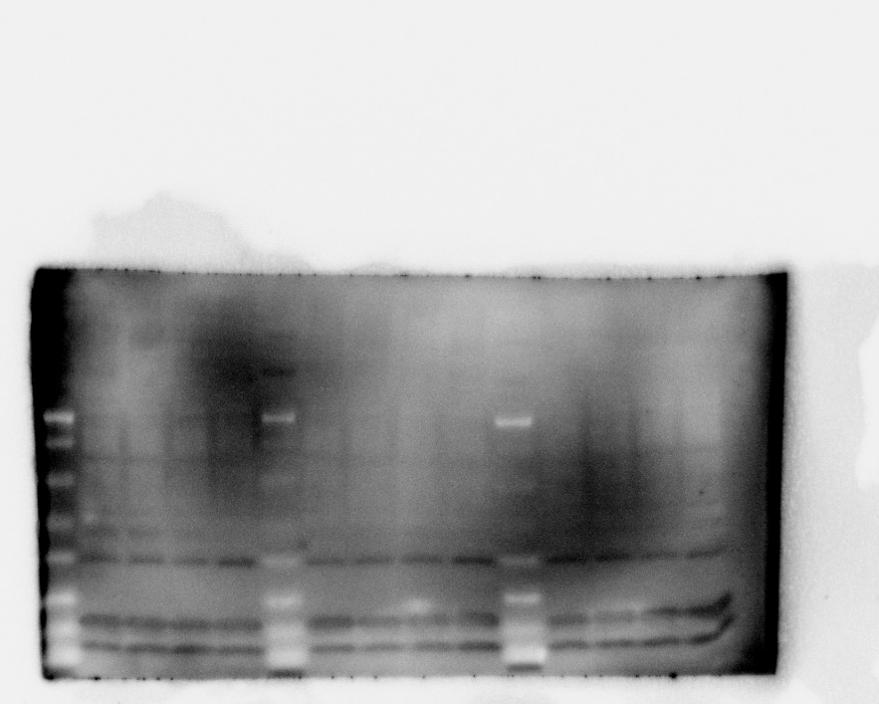
**

**1-β-Actin**

**
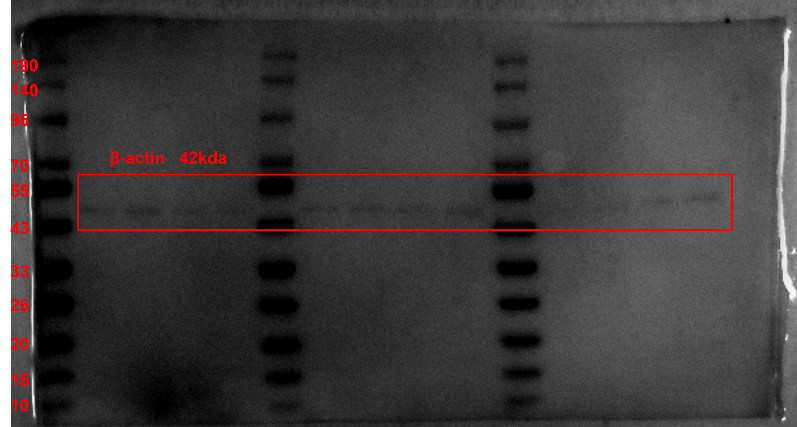

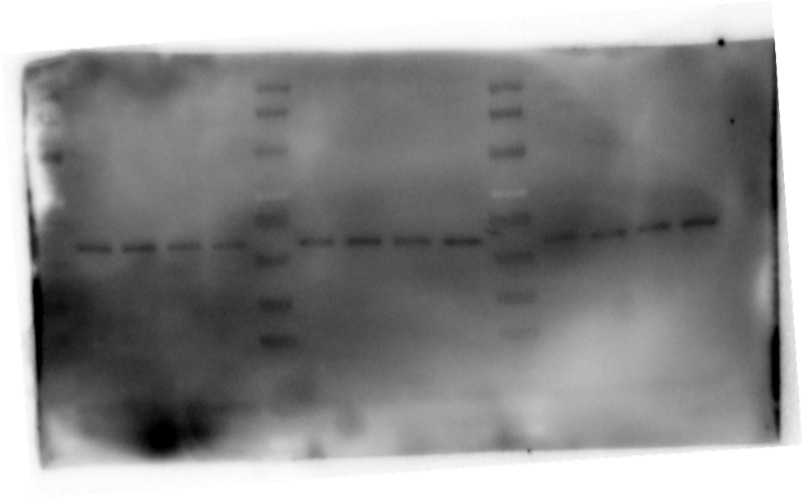
**

**
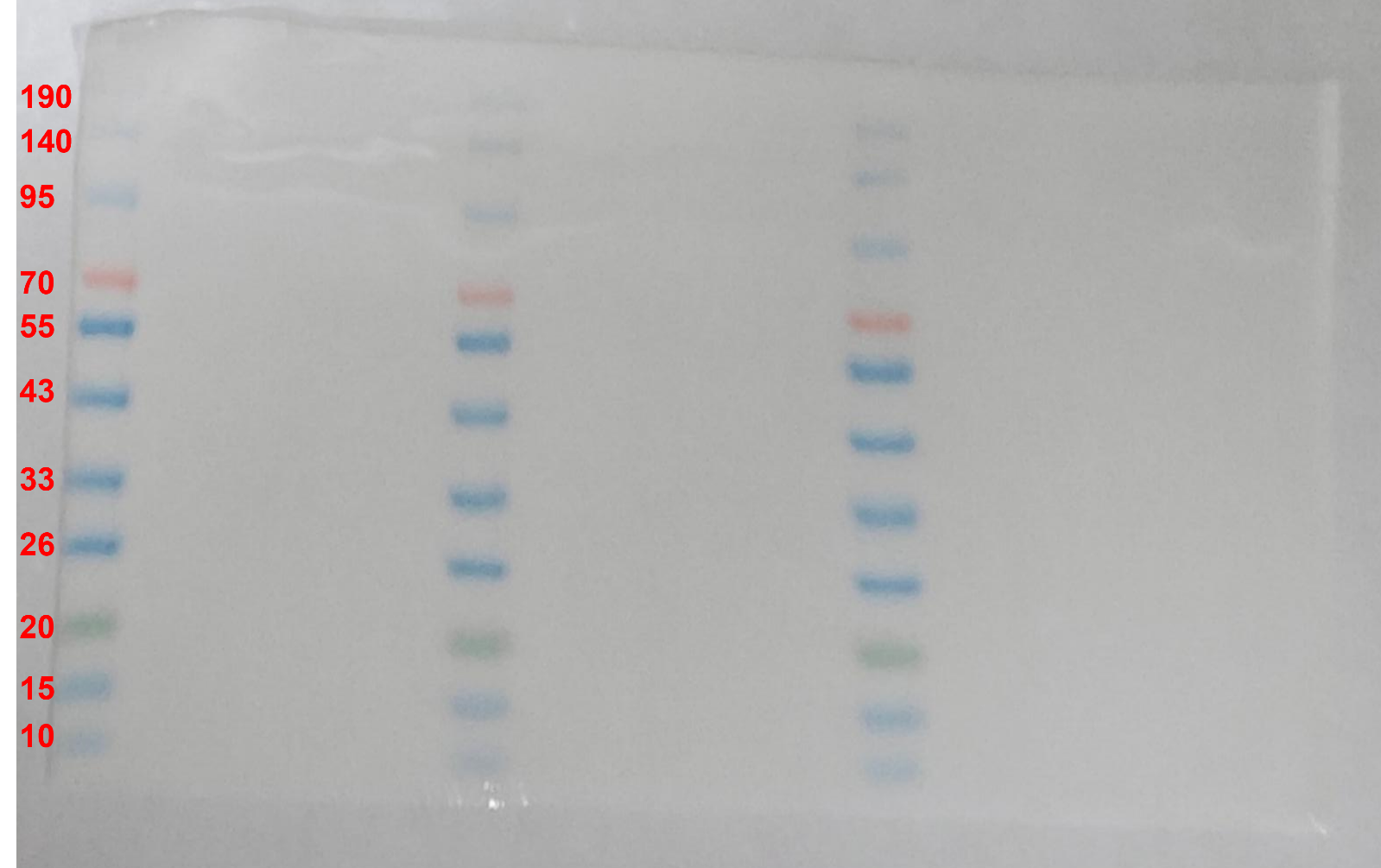
**

**2-BCL-xl**

**
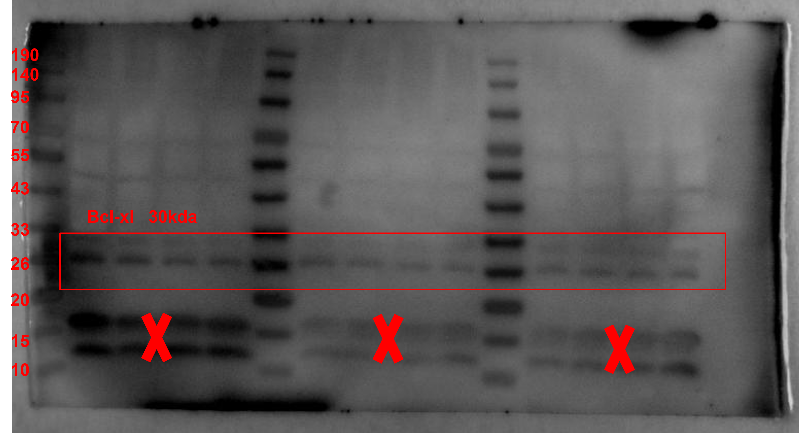

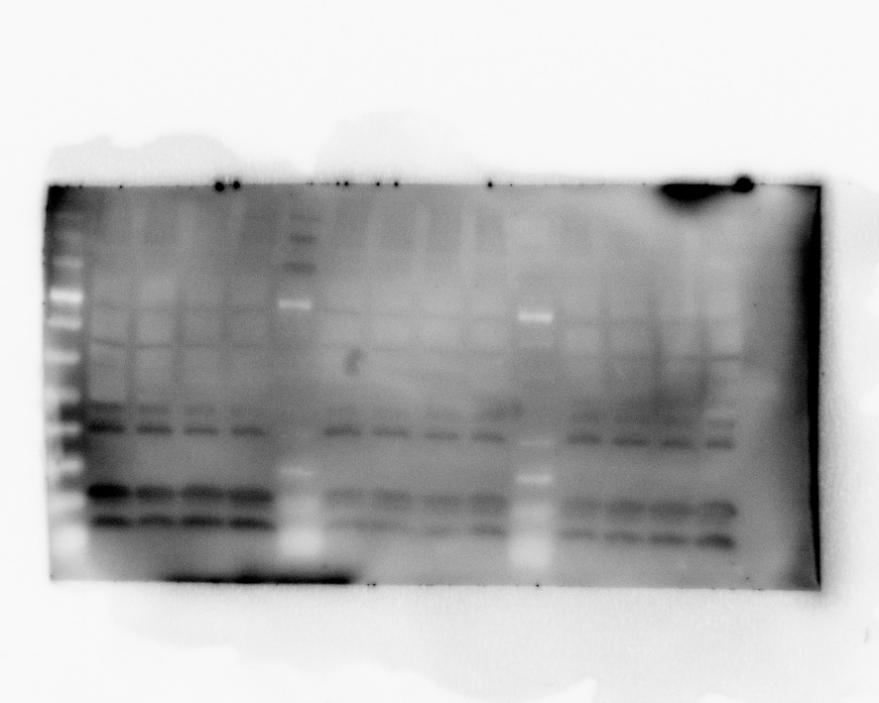
**

**2-β-Actin**

**
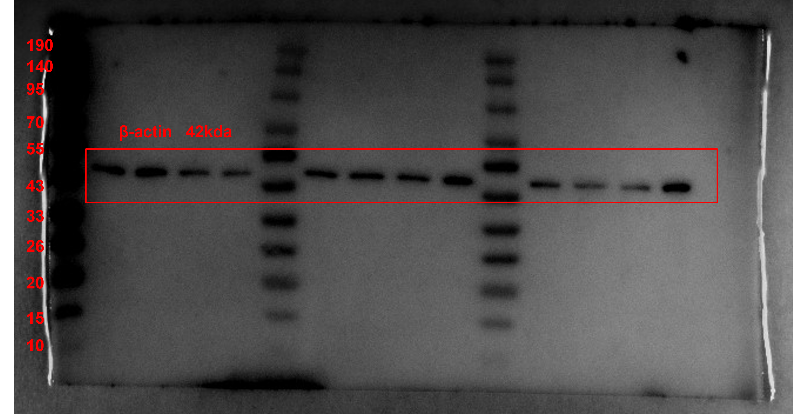

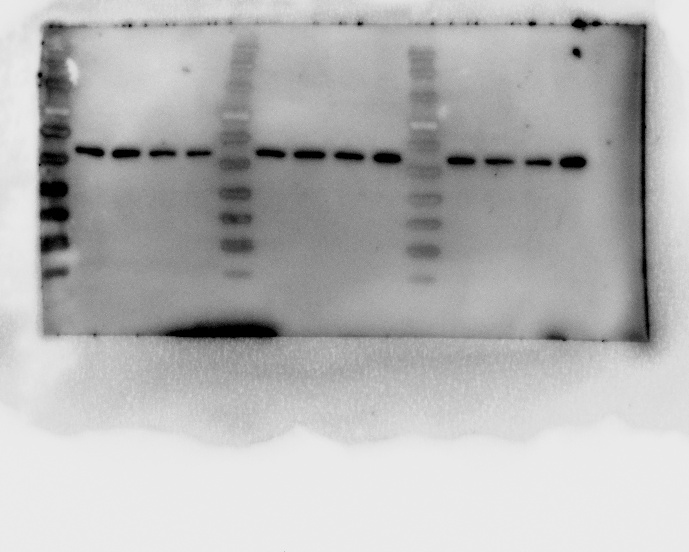
**

**
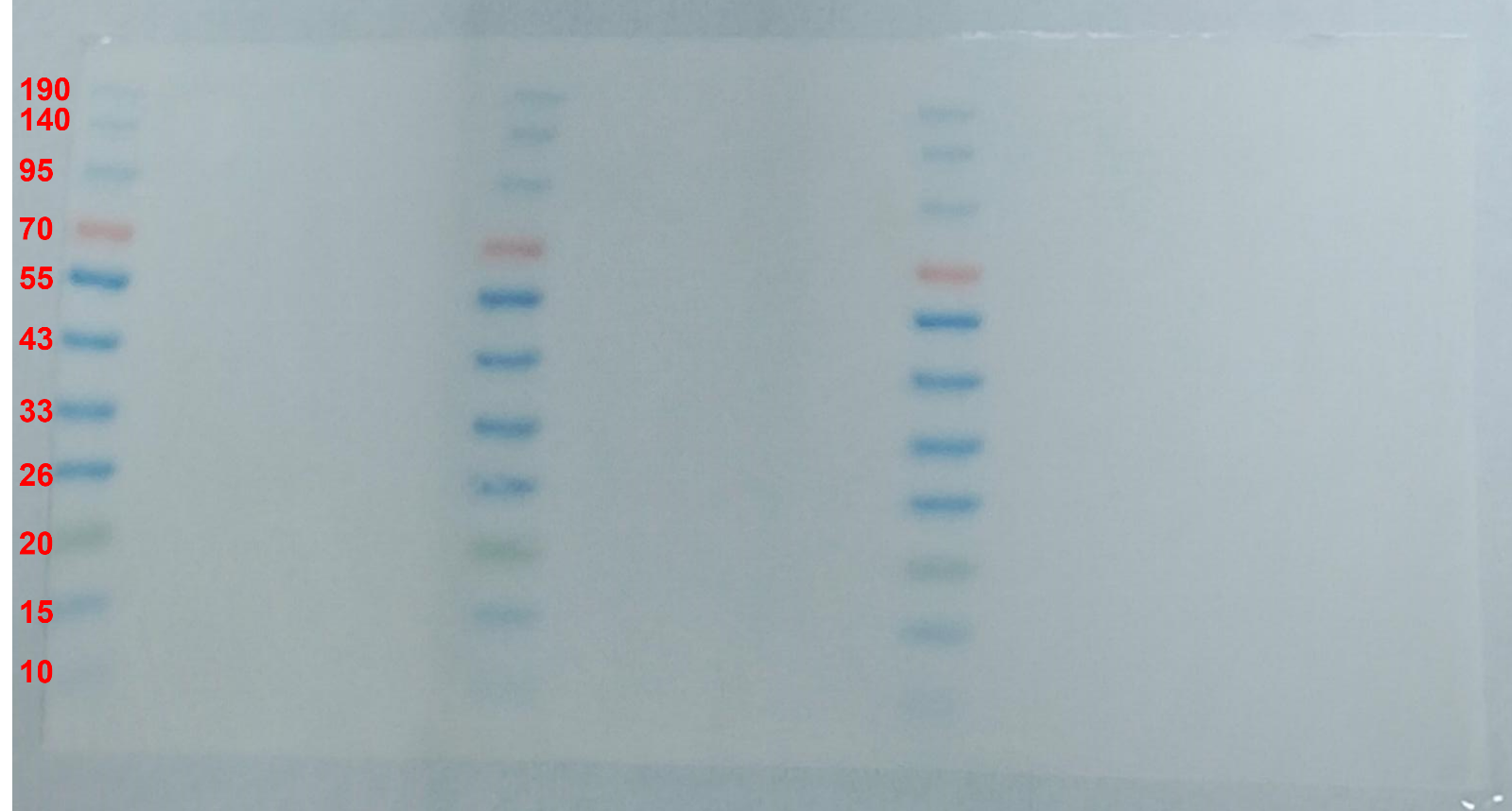
**

**3-Bax**


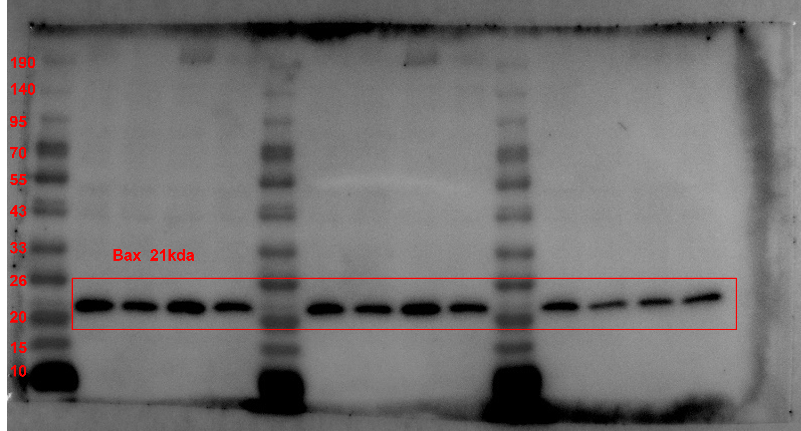

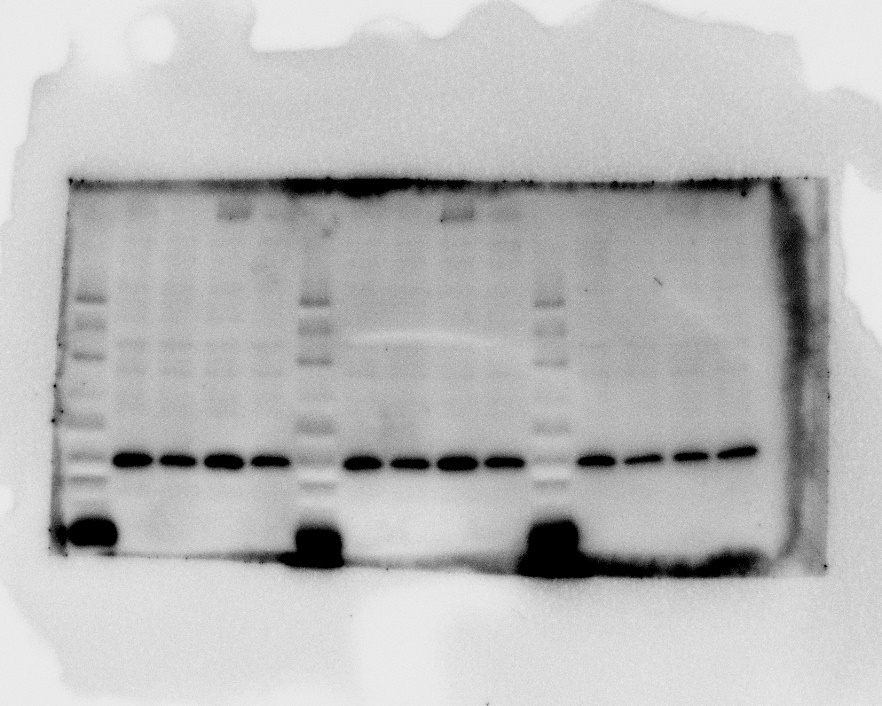


**3-β-Actin**


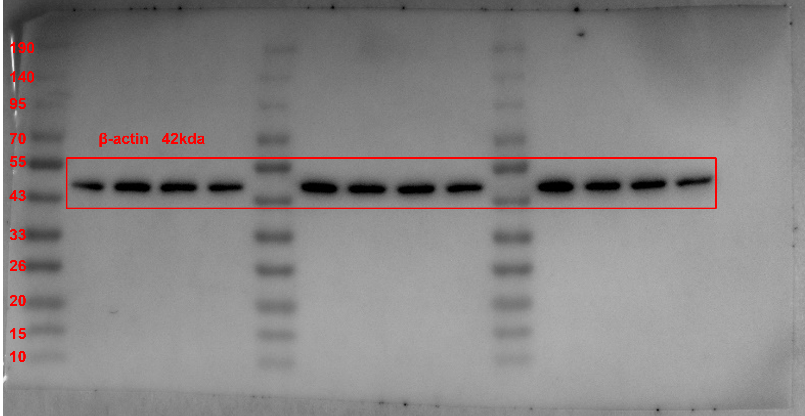

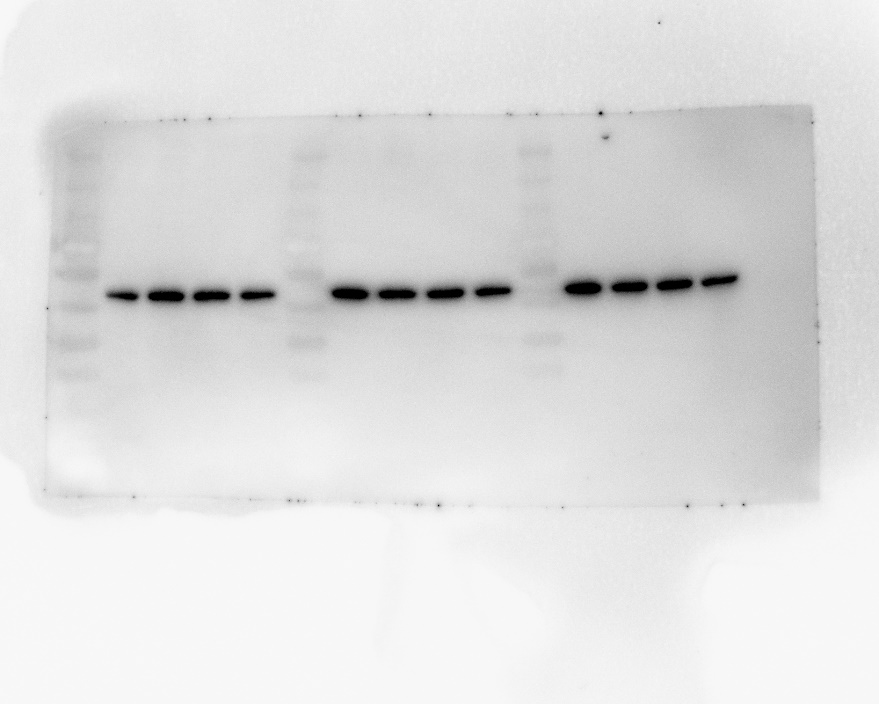


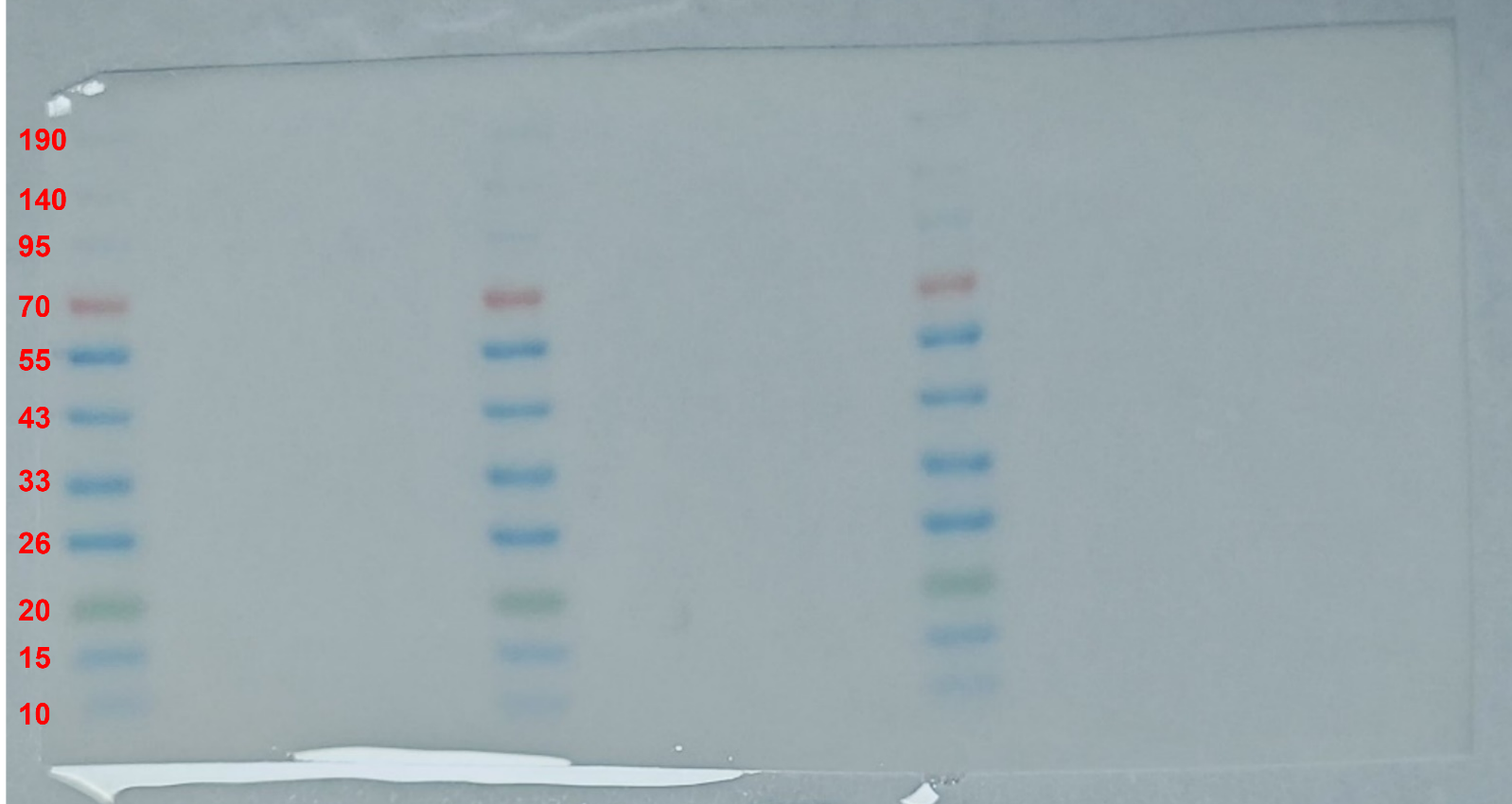


**4-Caspase3**

**
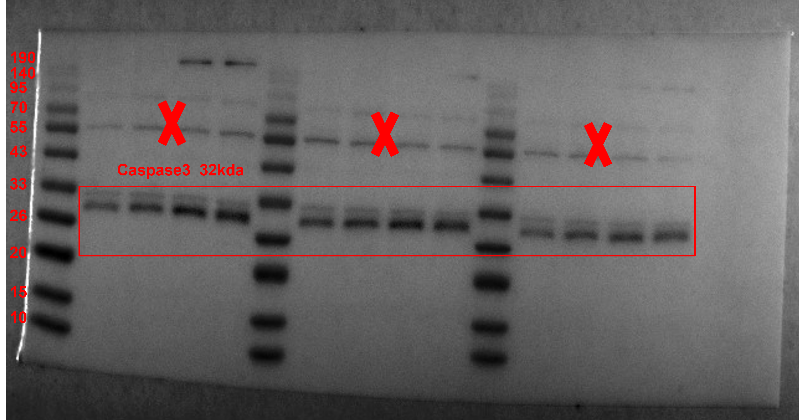

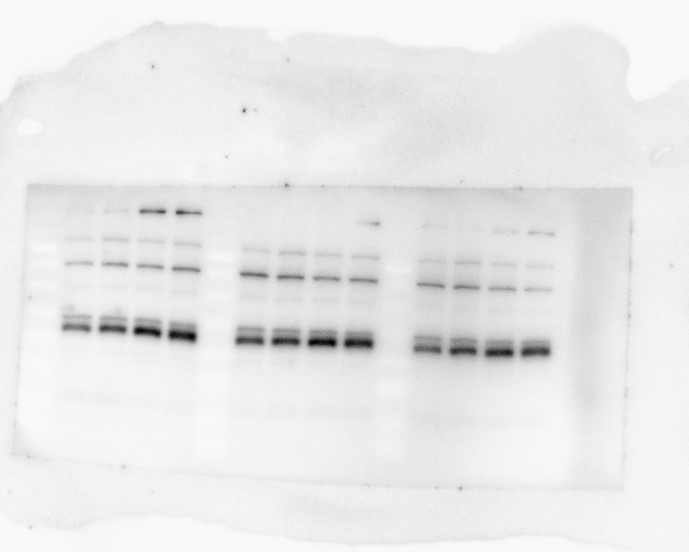
**

**4-β-Actin**

**
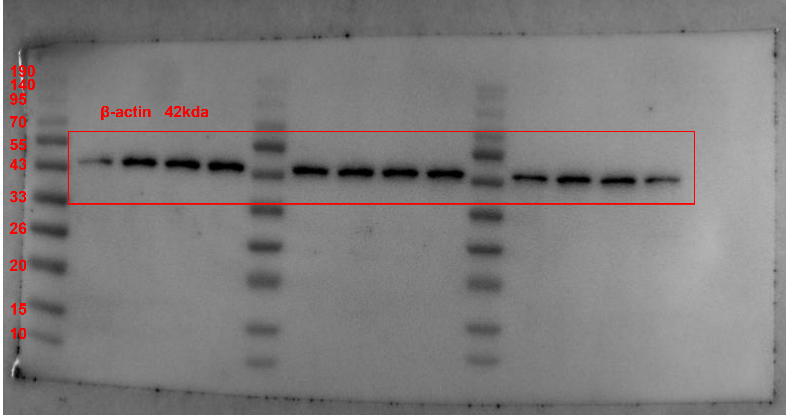

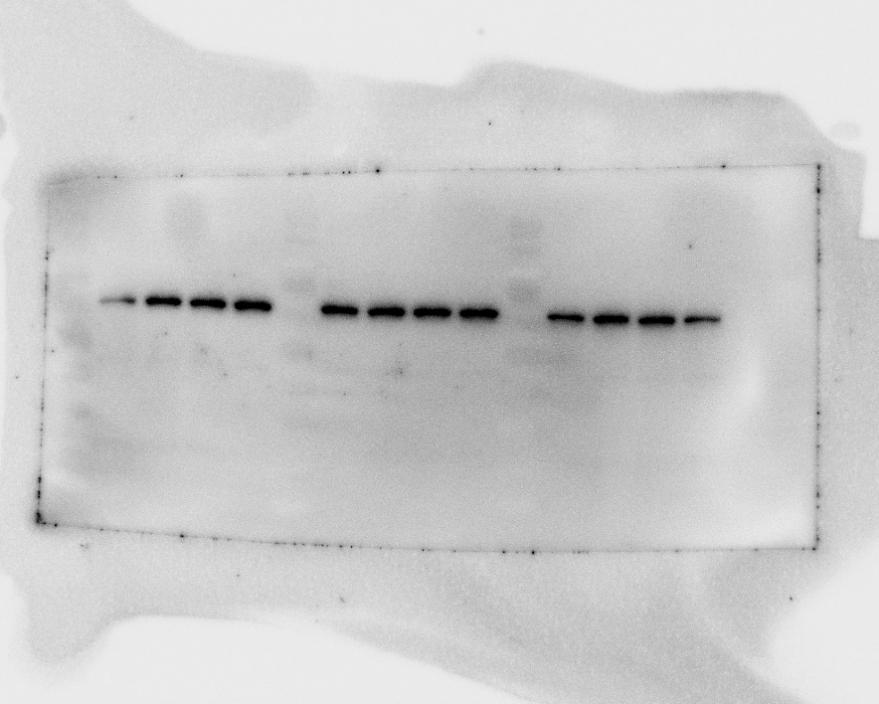
**

**
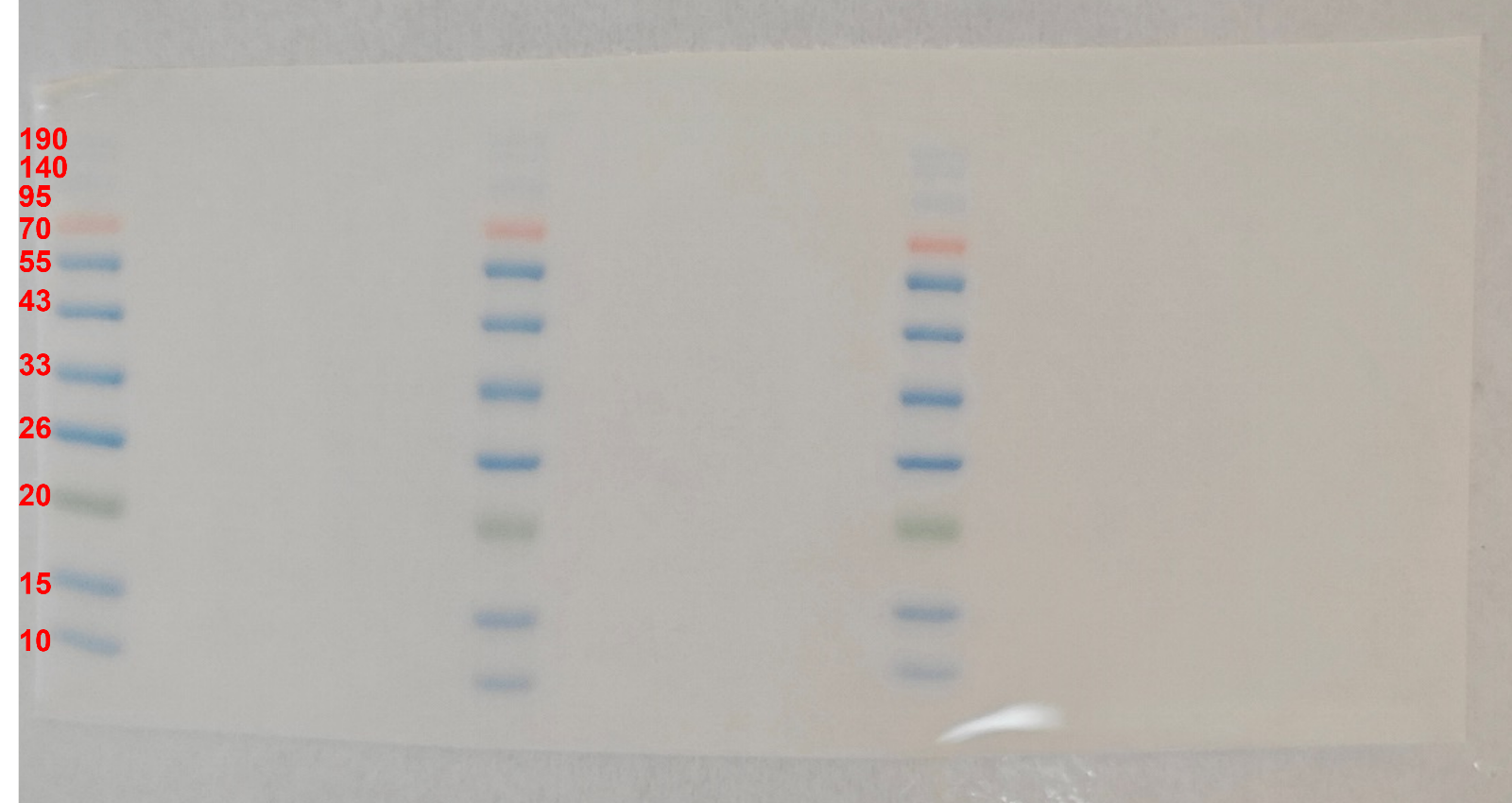
**

**5-Caspase9**


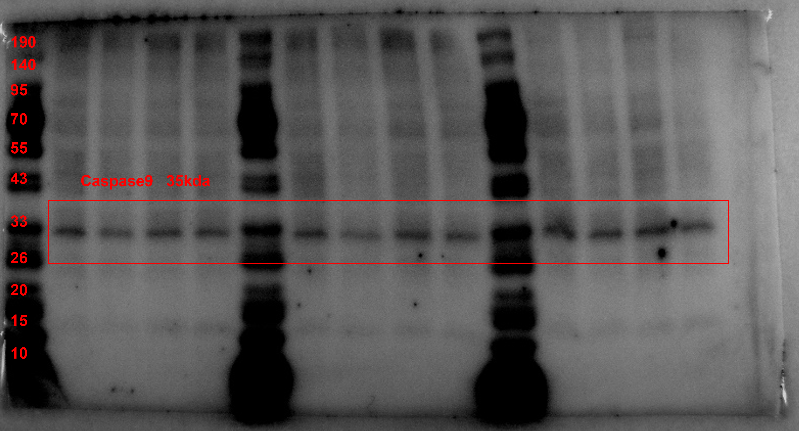

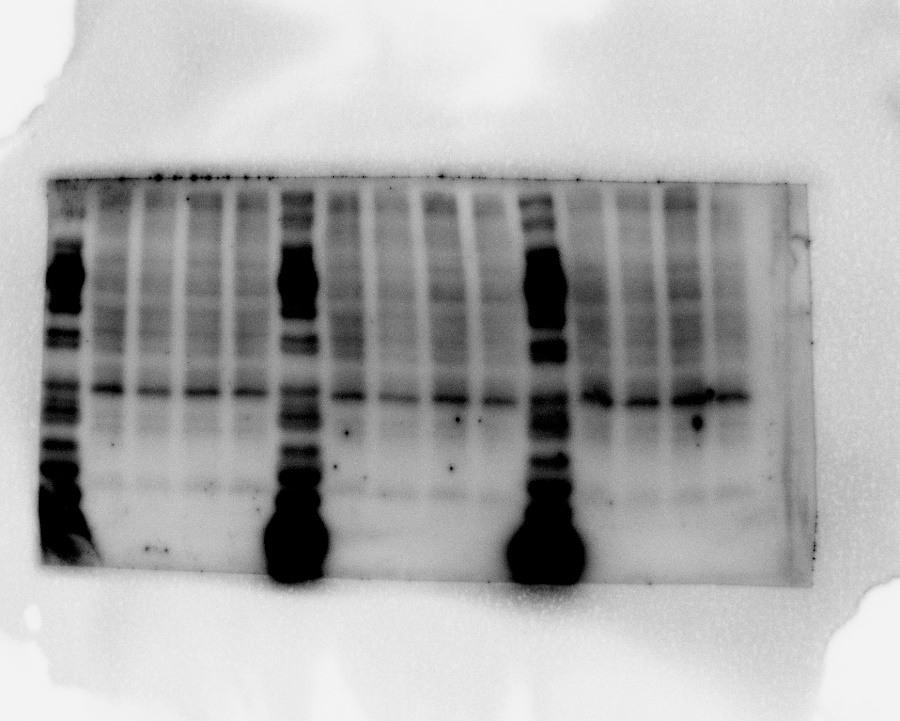


**5-β-Actin**


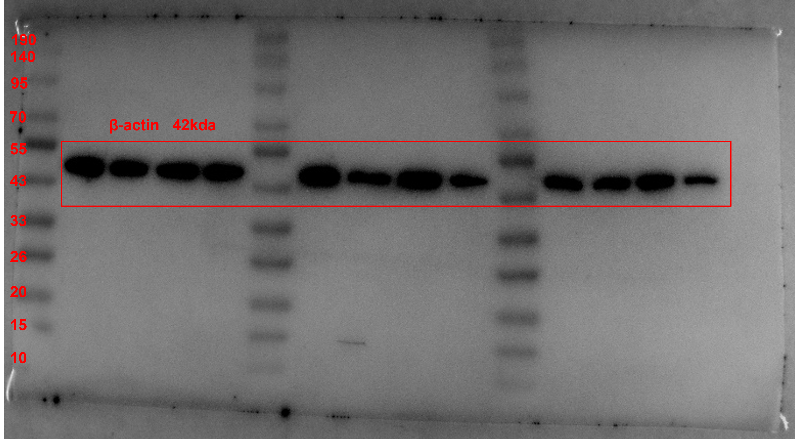

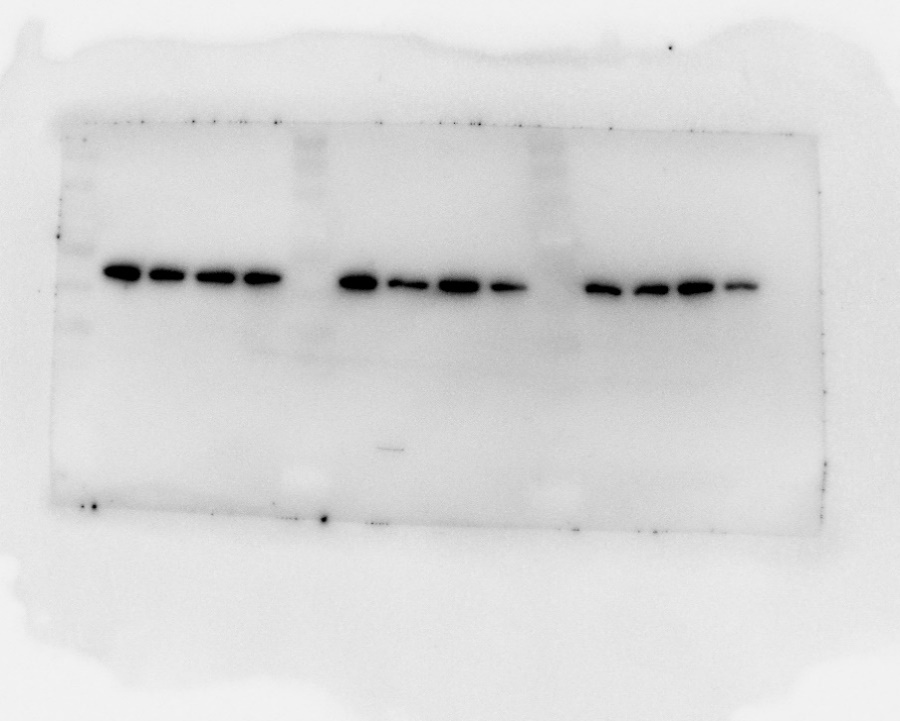


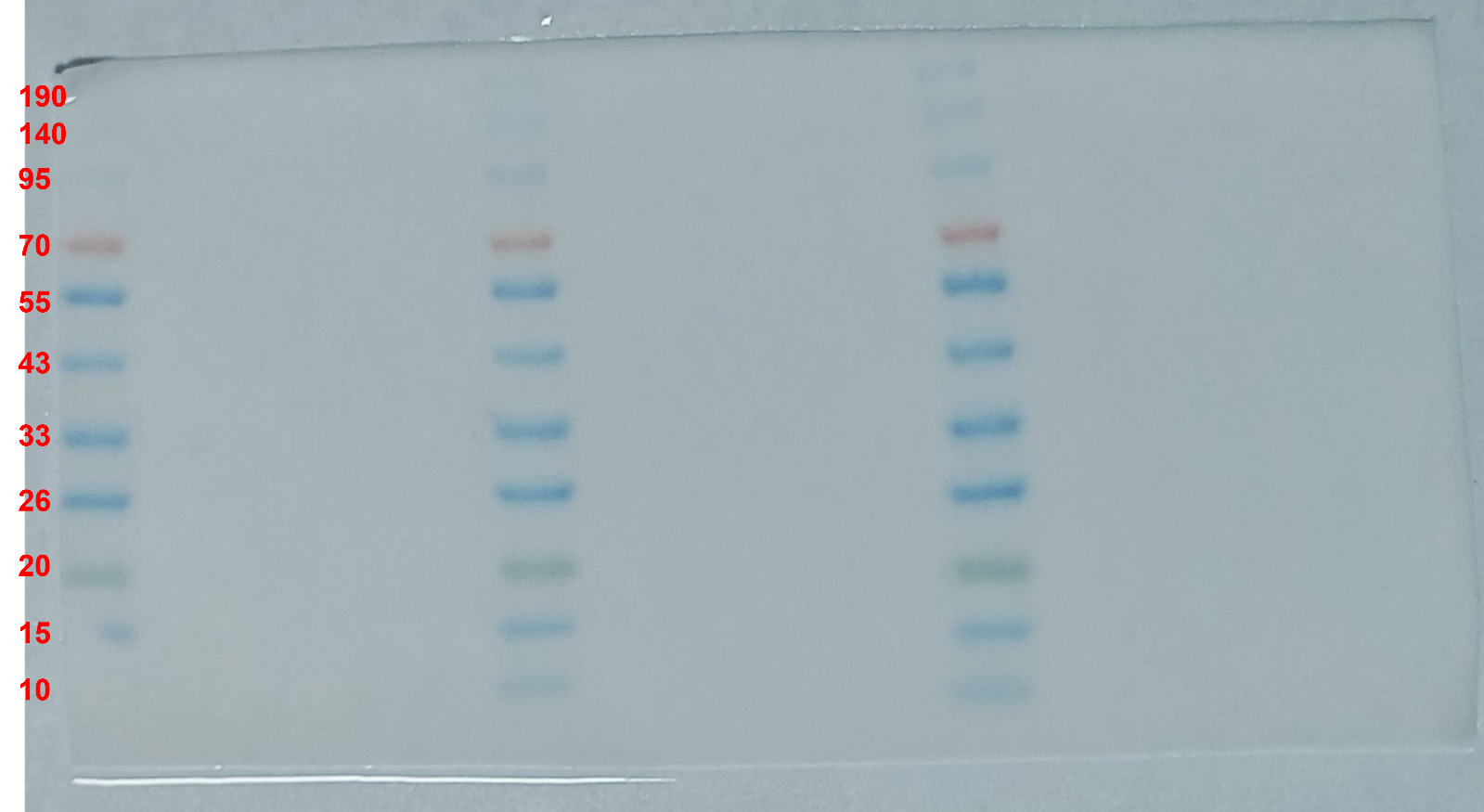


**6-Keap1**

**
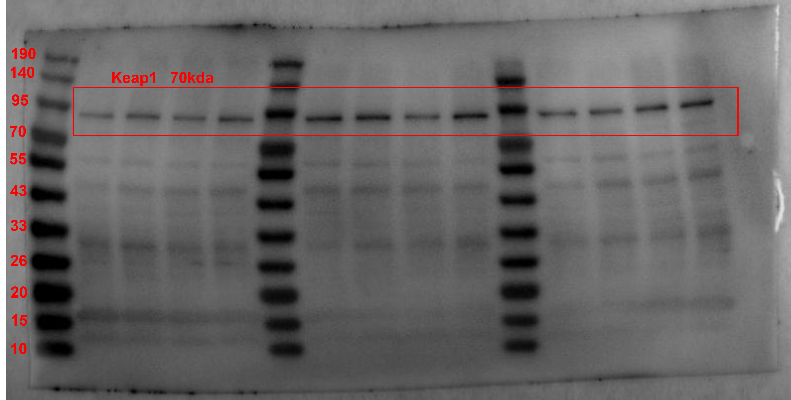

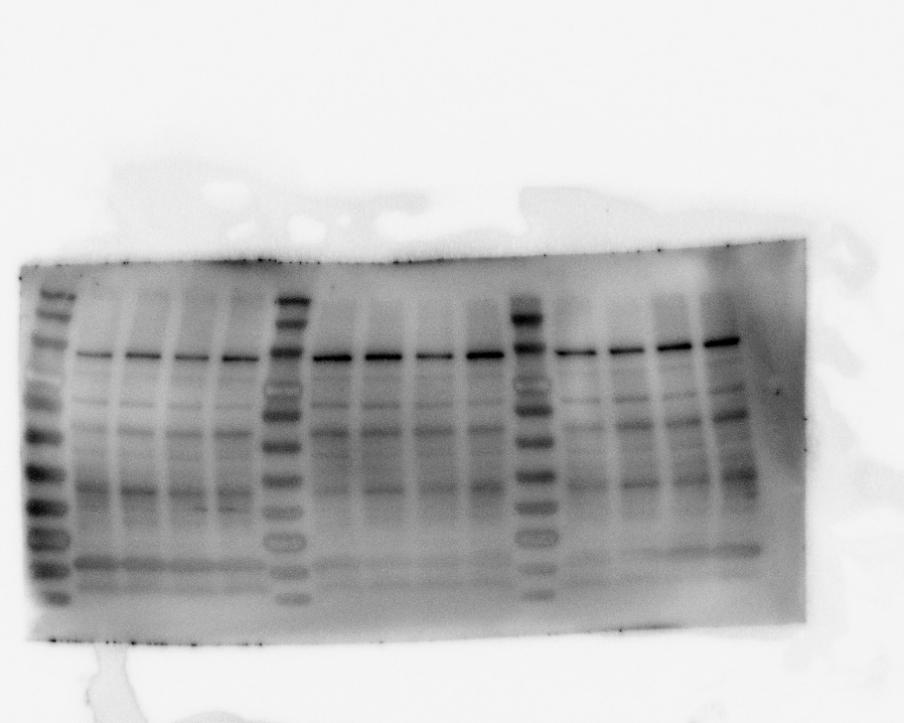
**

**6-β-Actin**

**
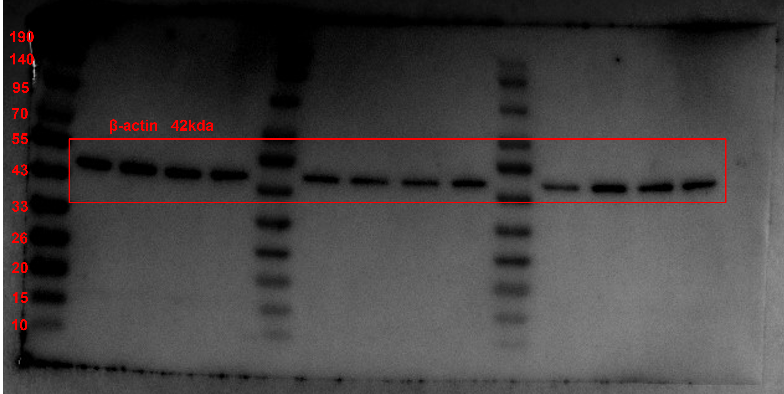

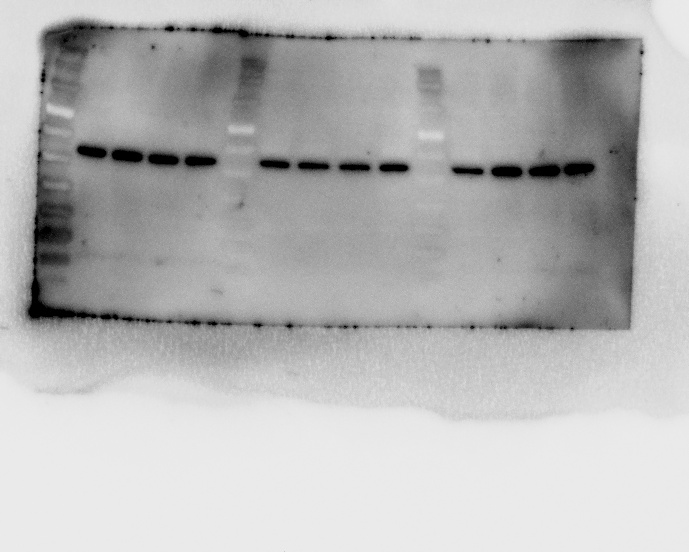
**

**
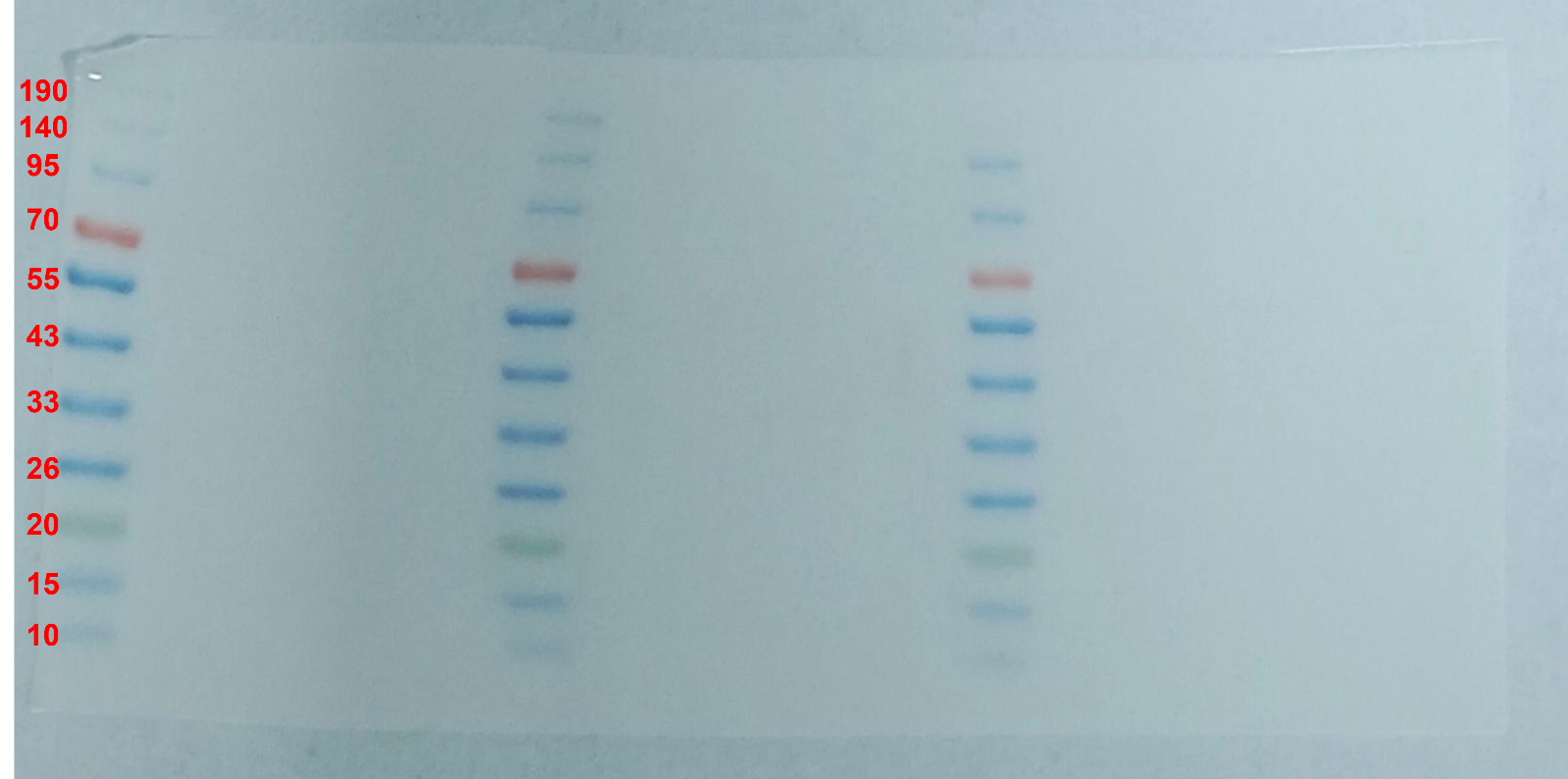
**

**7-NQO1**

**
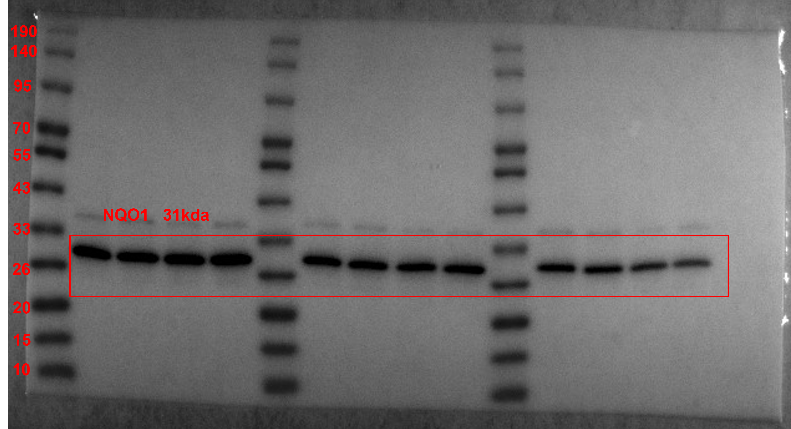

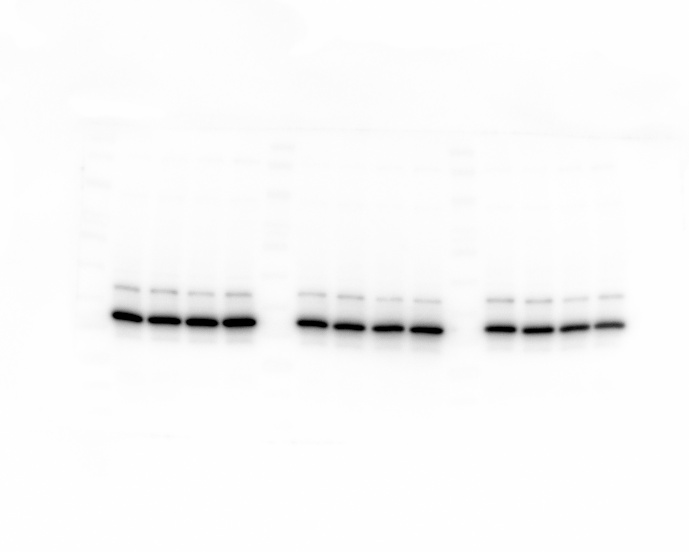
**

**7-β-Actin**

**
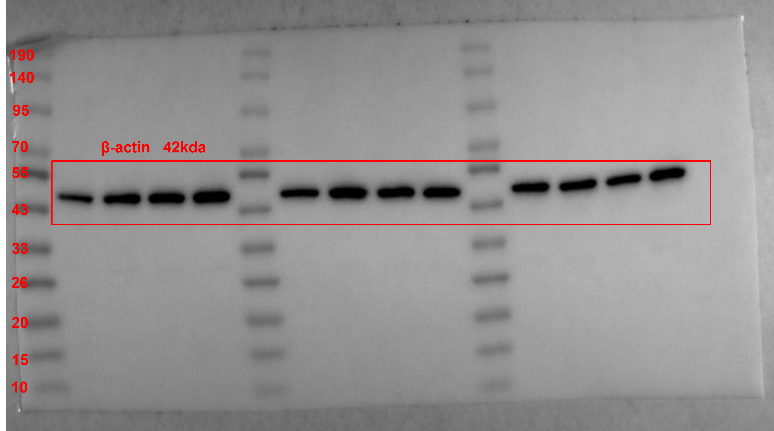

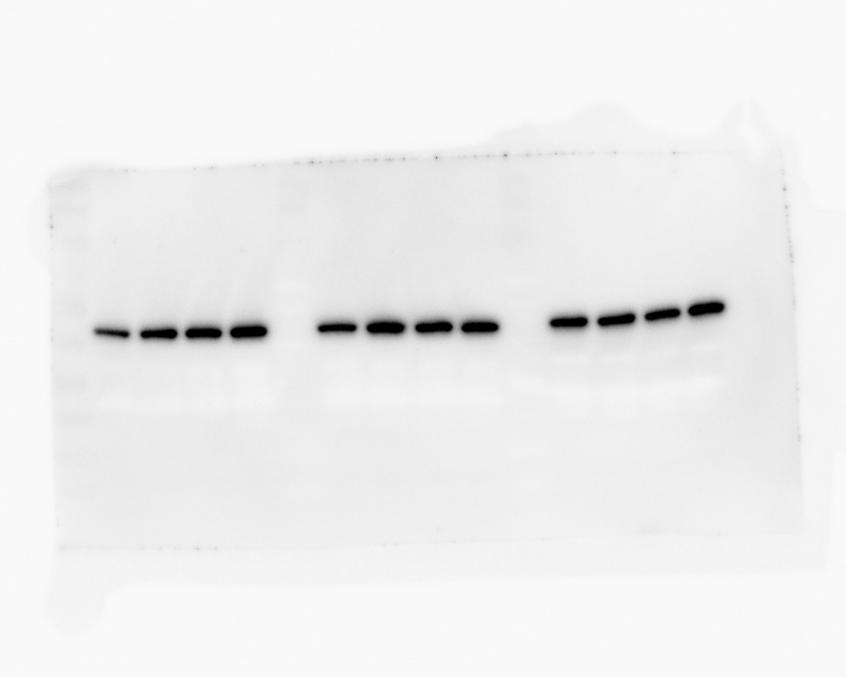
**

**
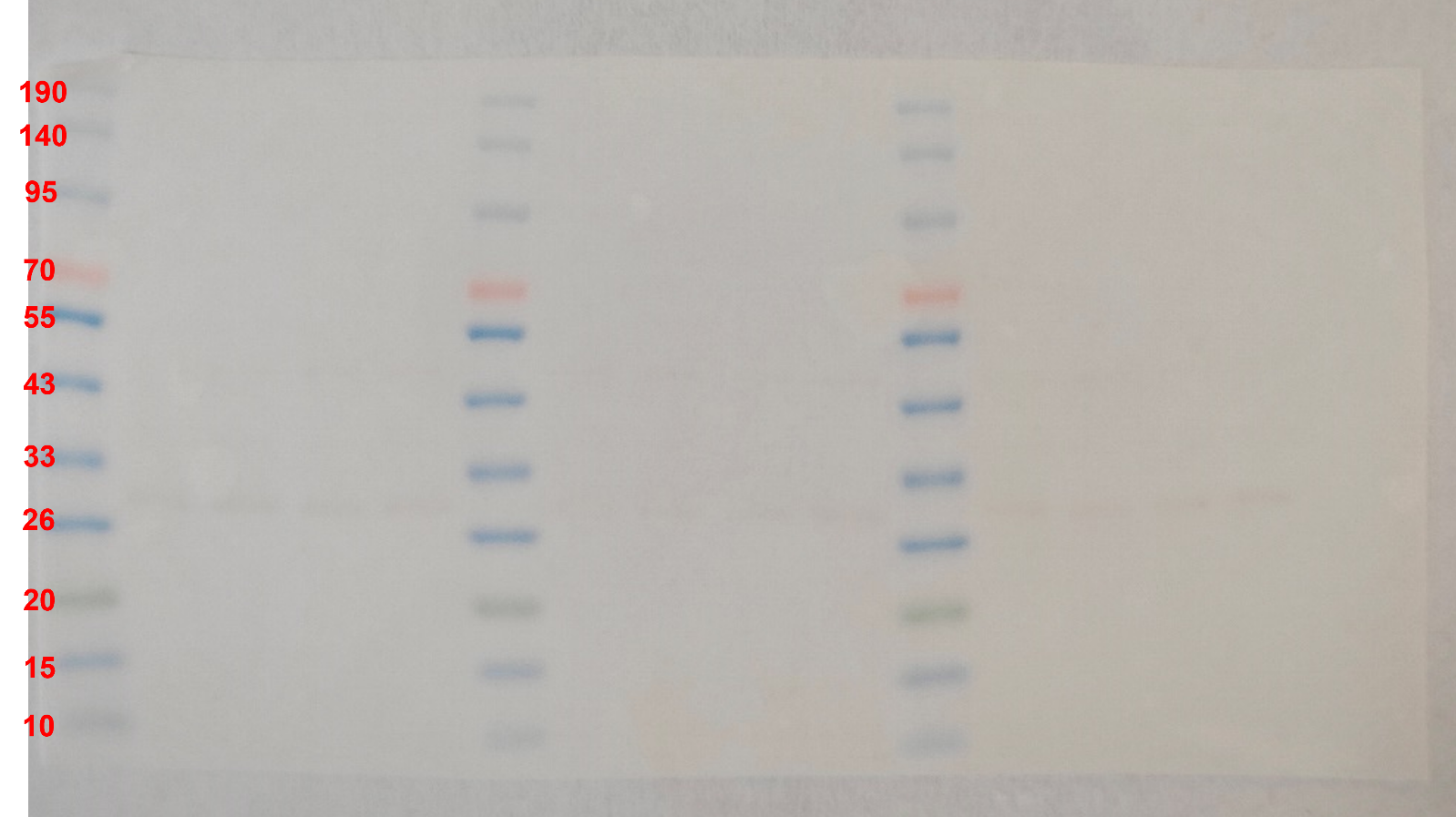
**

**8-HO-1**

**
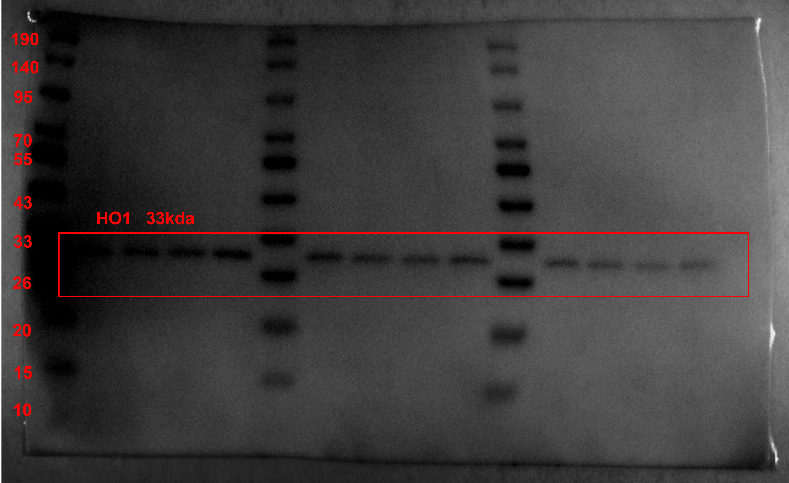

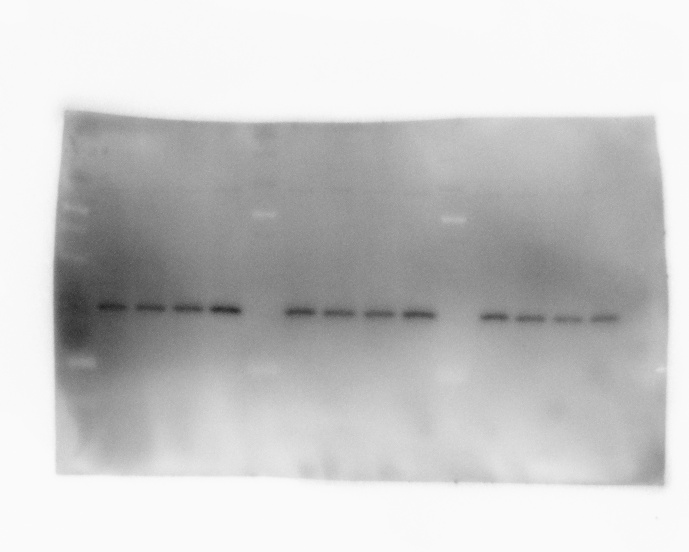
**

**8-β-Actin**

**
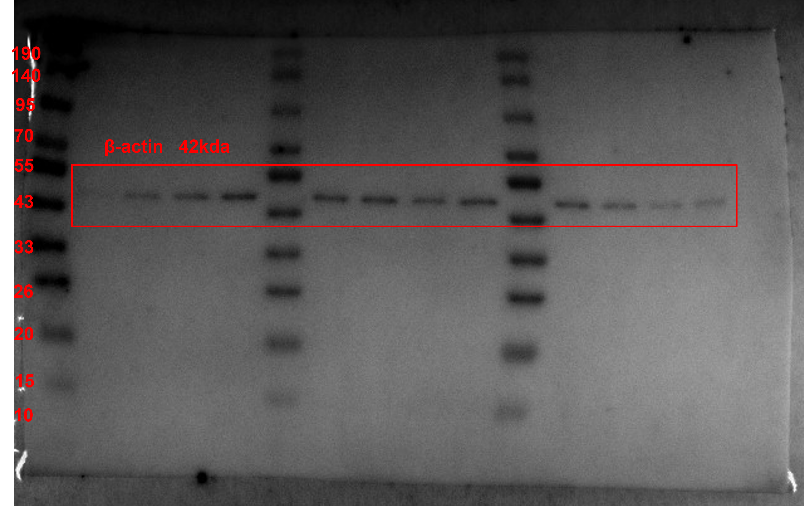

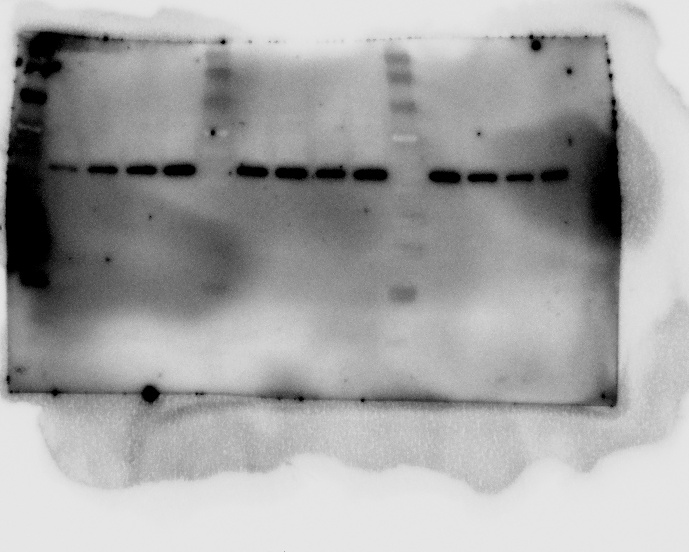
**

**
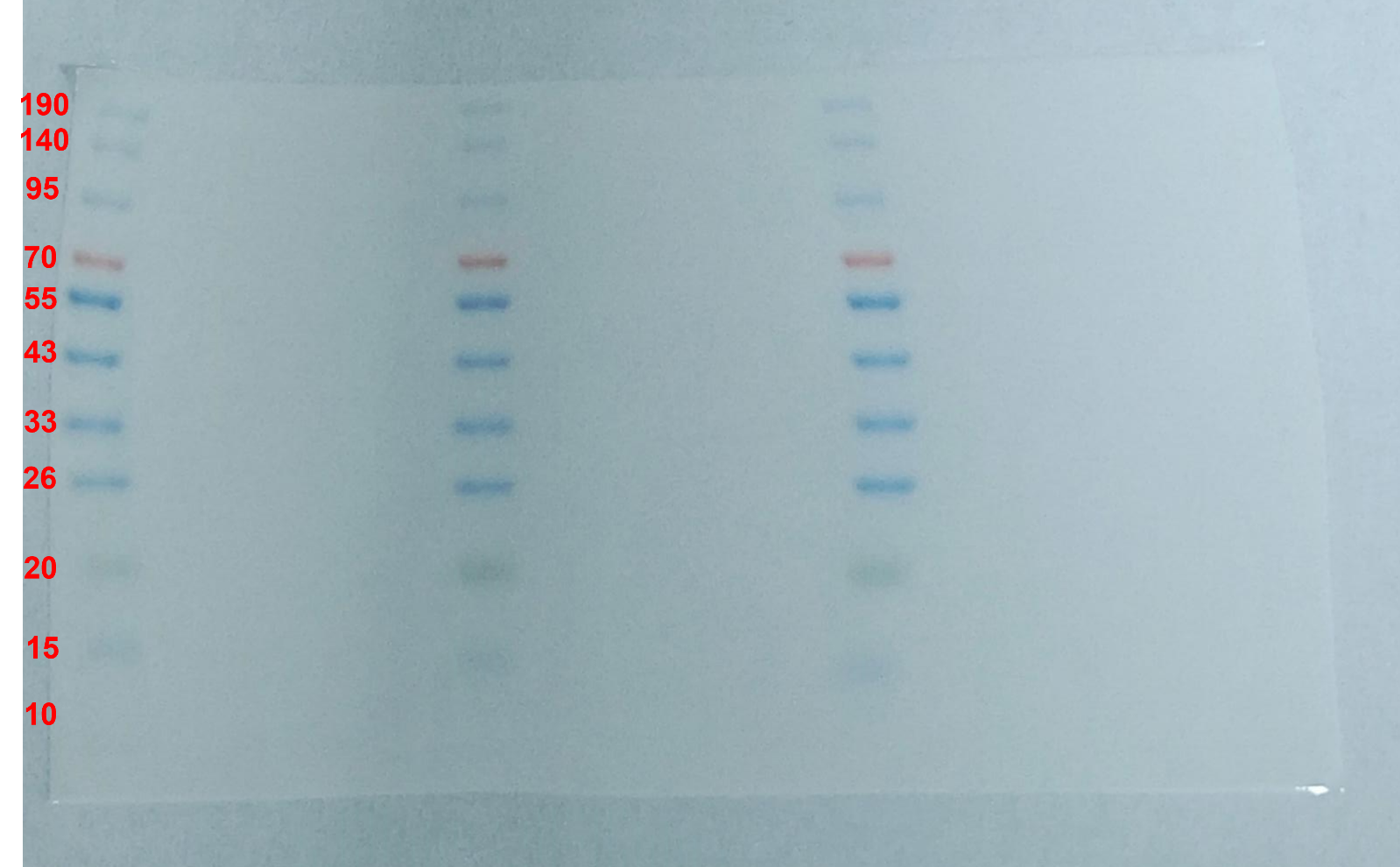
**

**9-Nrf2**

**
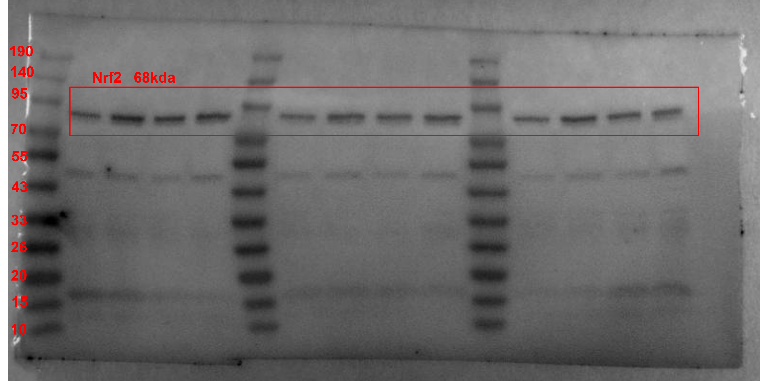

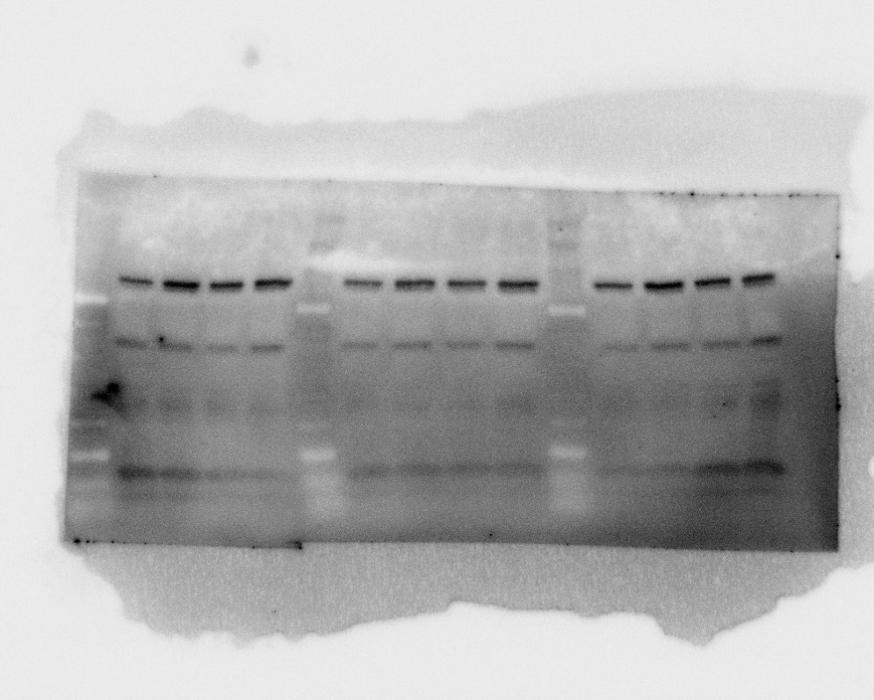
**

**9-β-Actin**

**
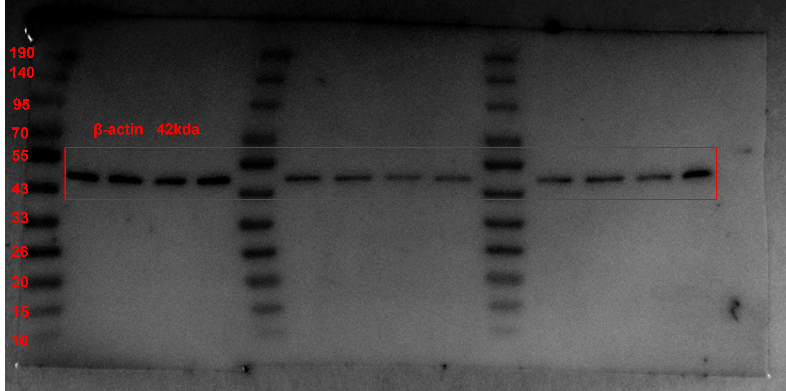

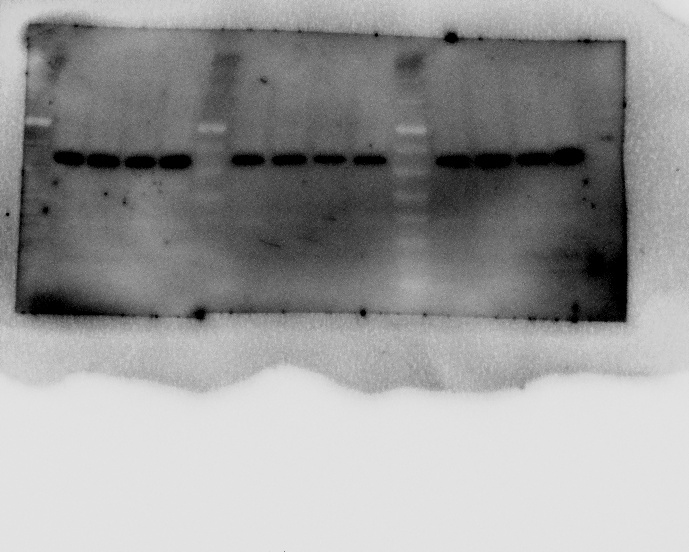
**

**
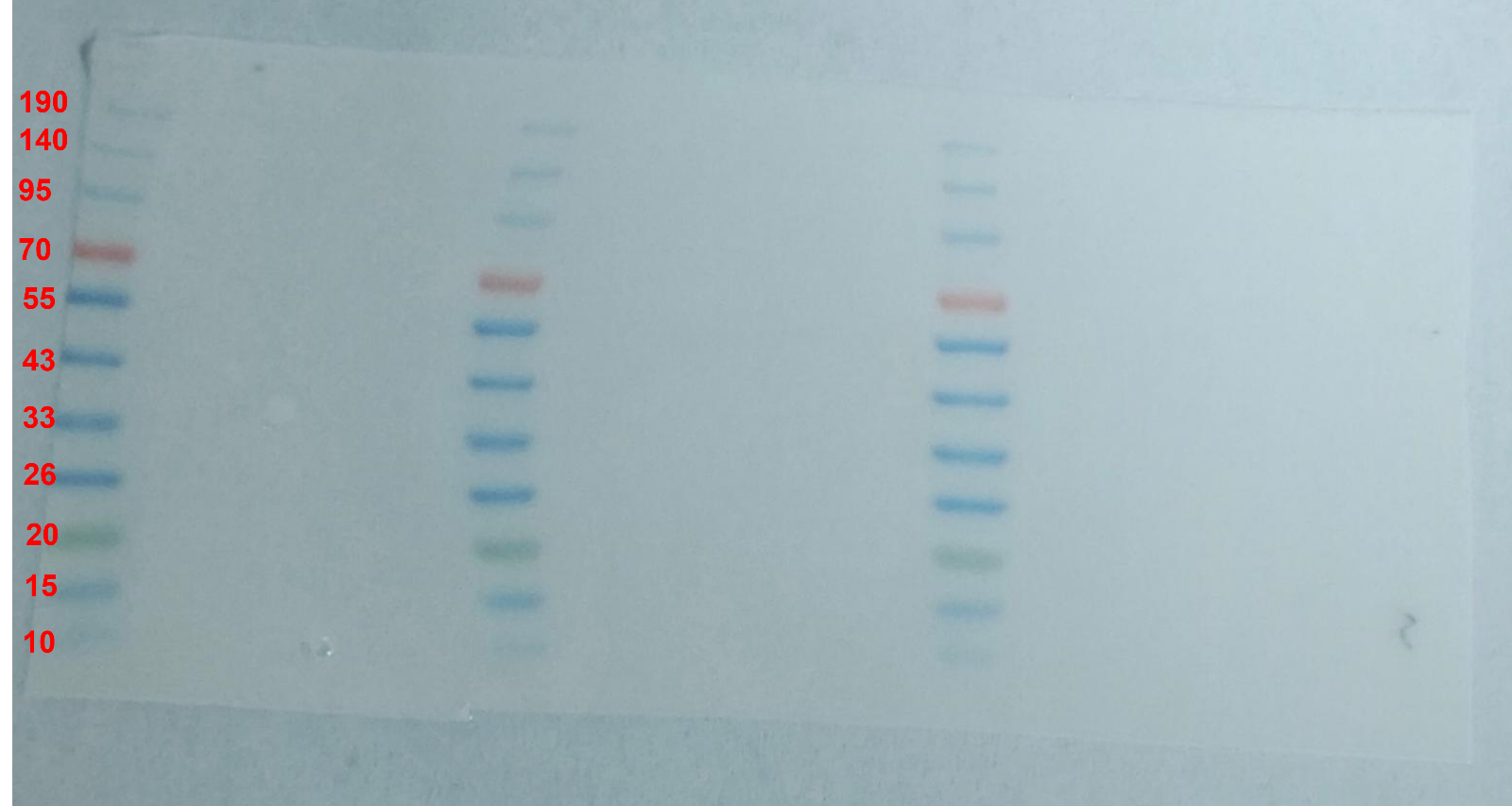
**

**10-ATP5F1**


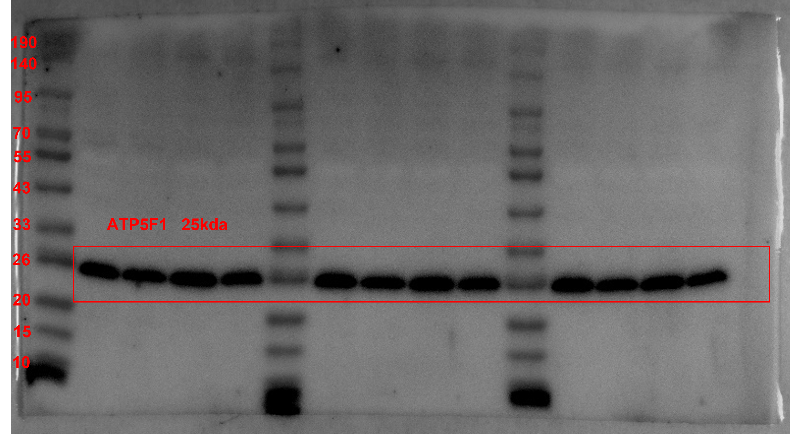

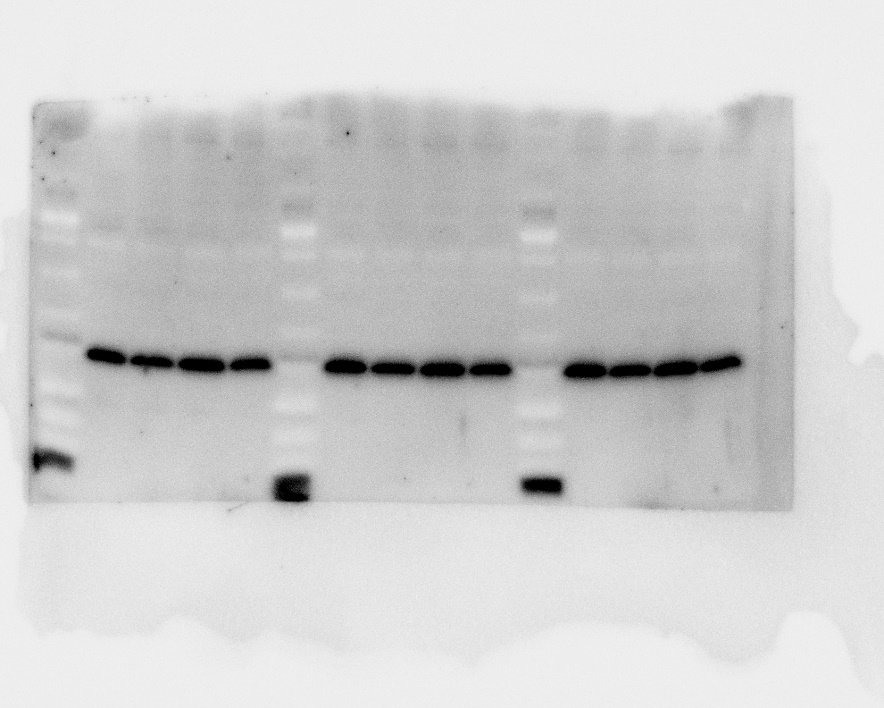


**10-COX4**


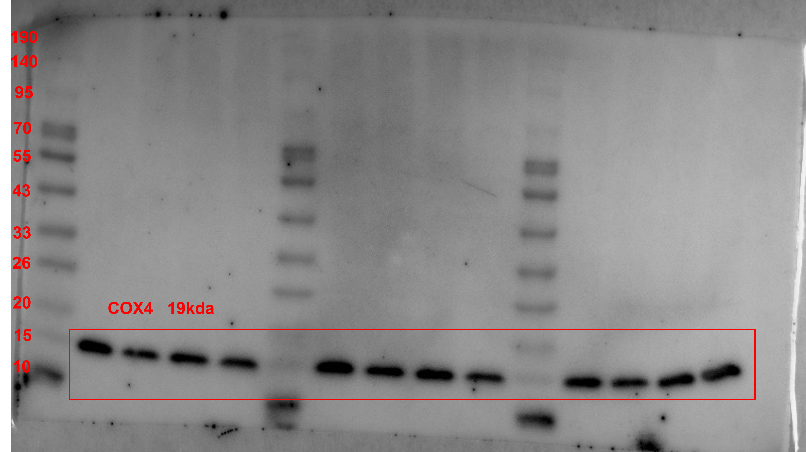

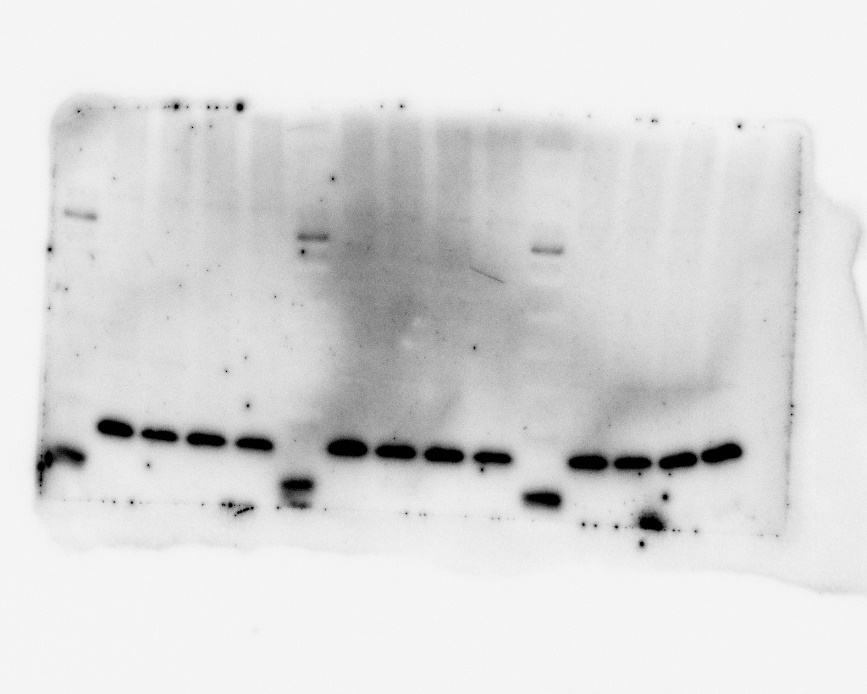


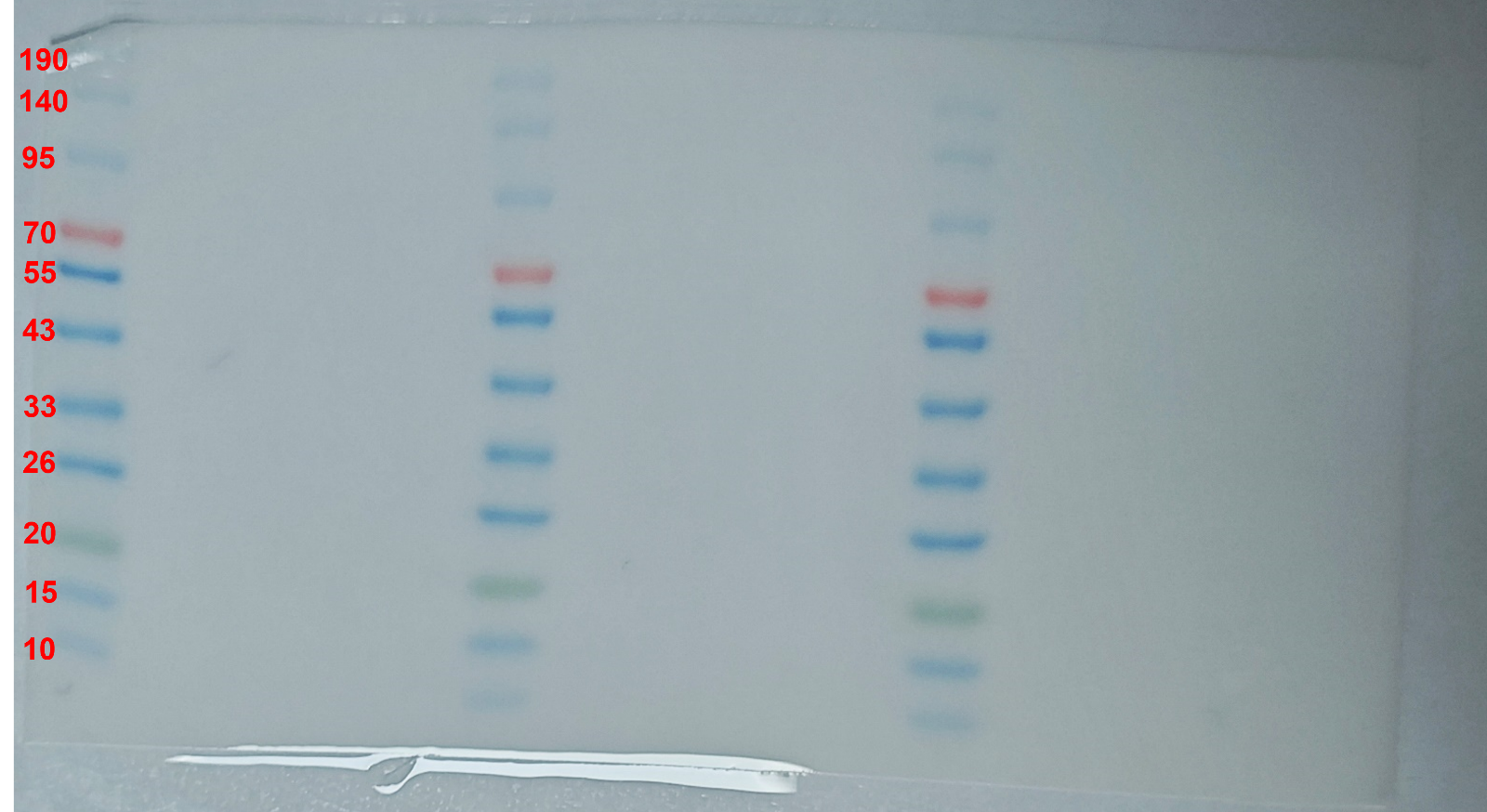


**11-CYC1**

**
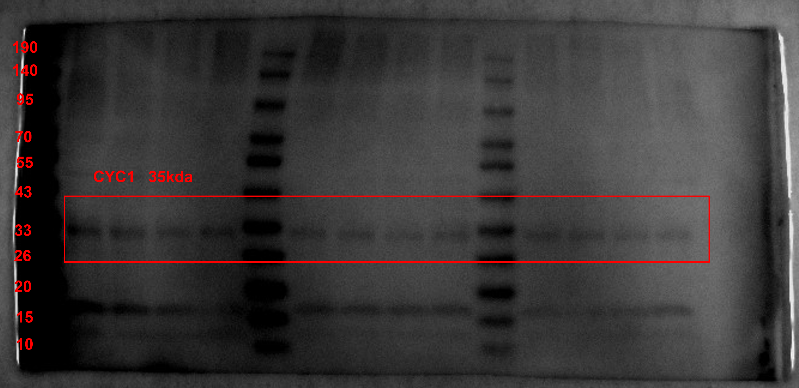

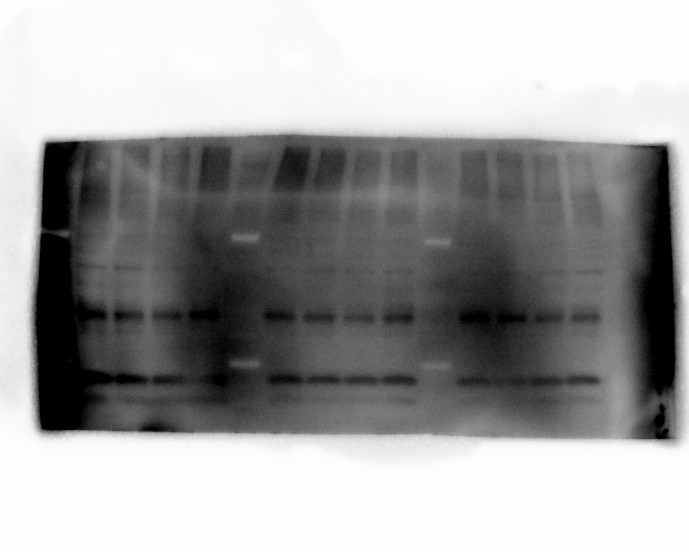
**

**11-Cox4**

**
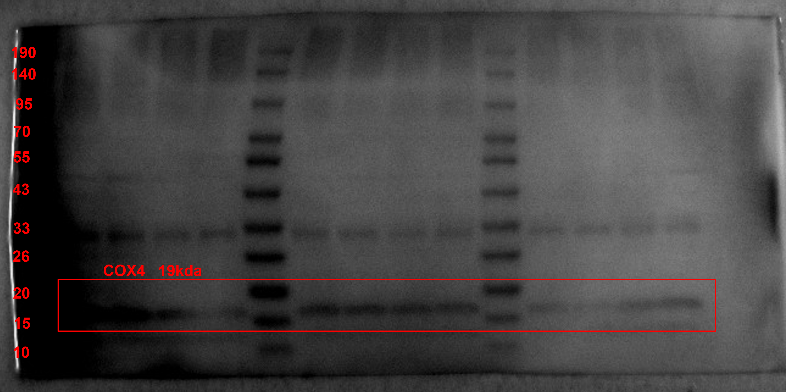

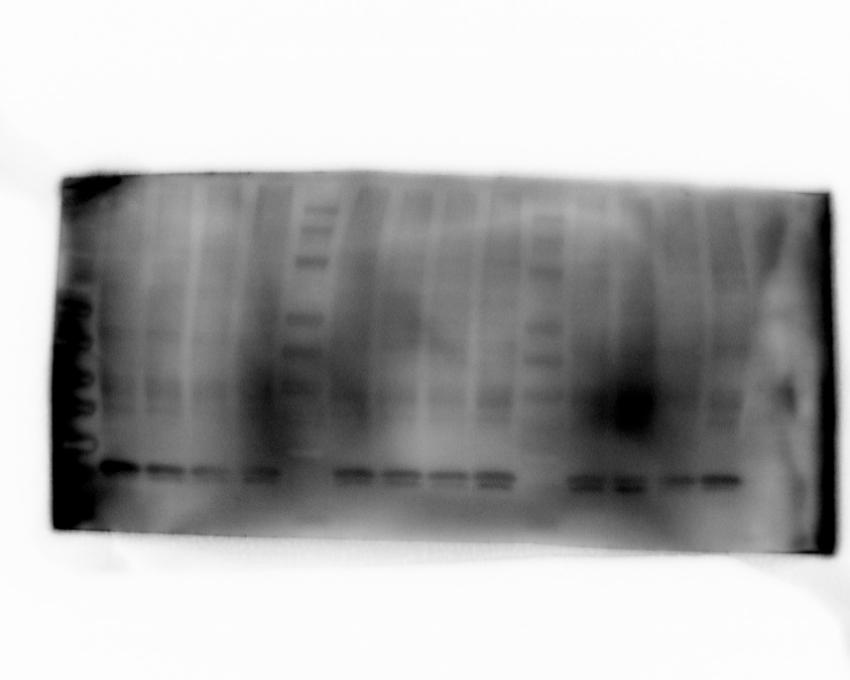
**

**
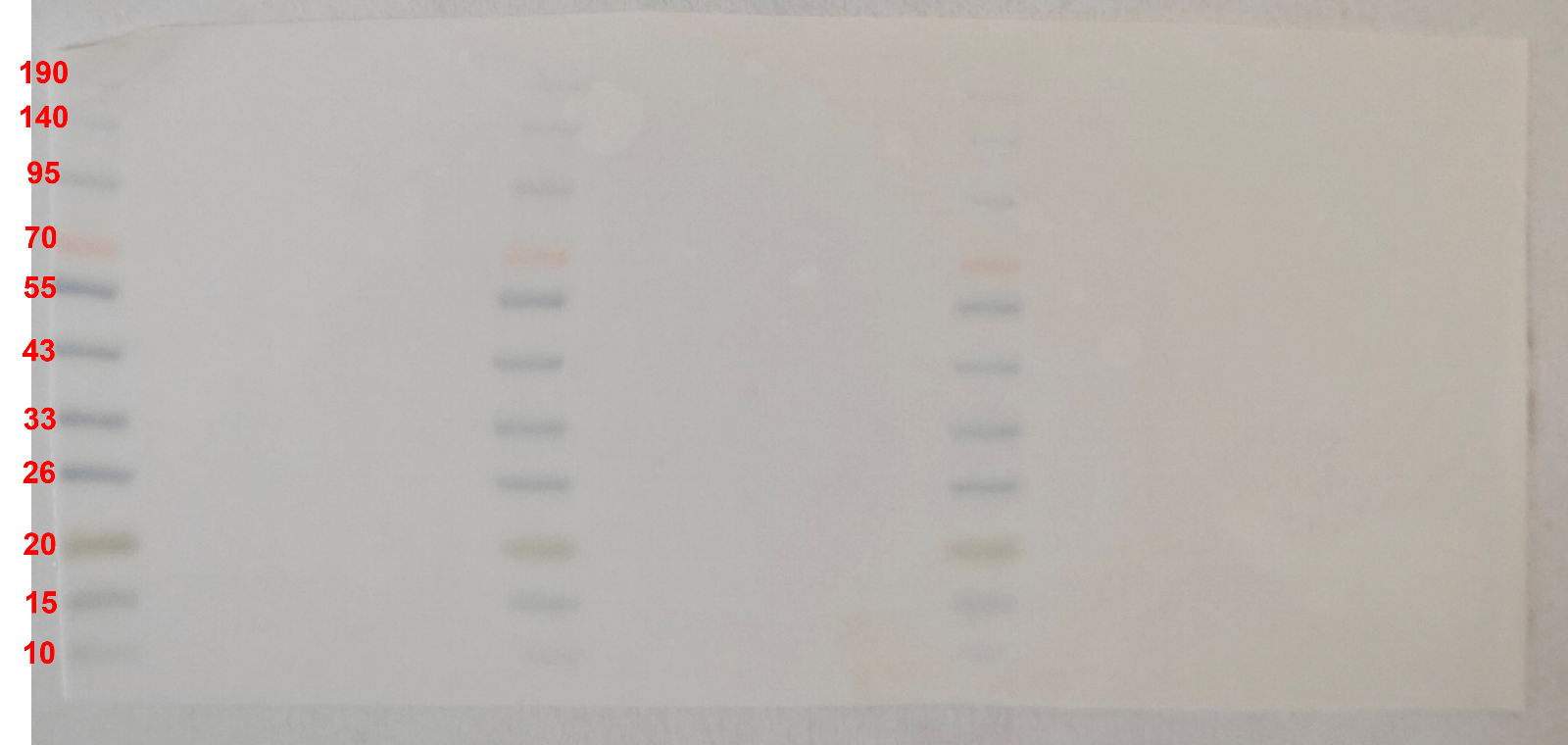
**

**12-NDUFV1**


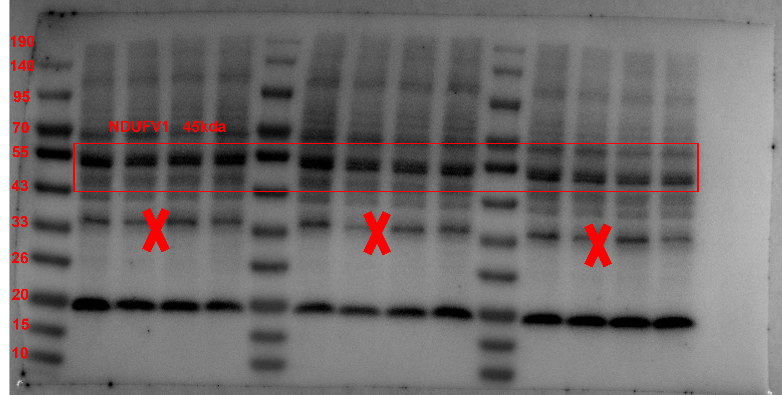

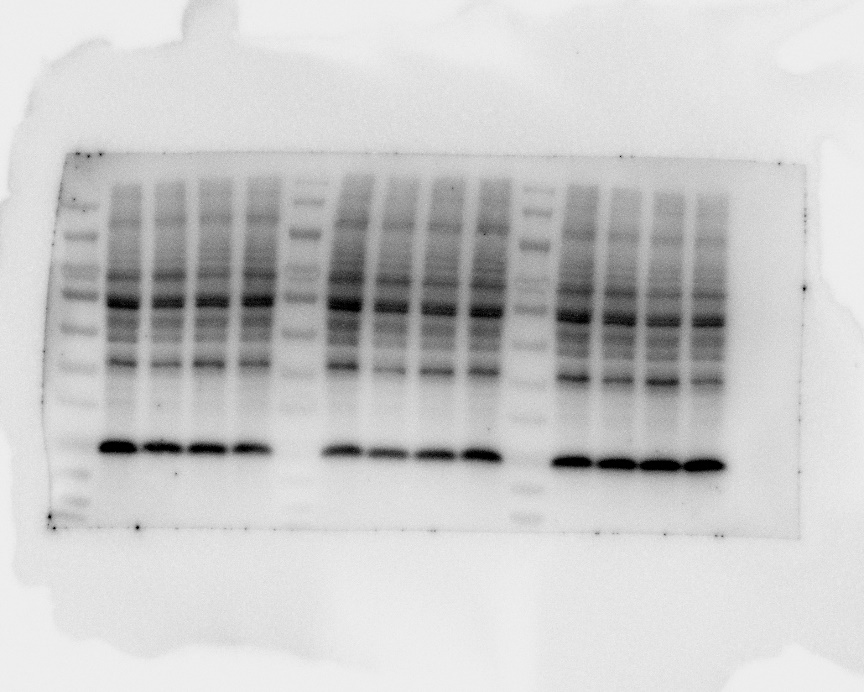


**12-Cox4**


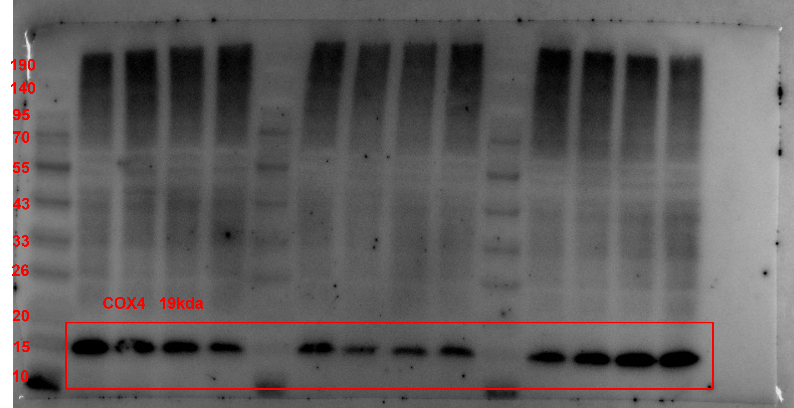

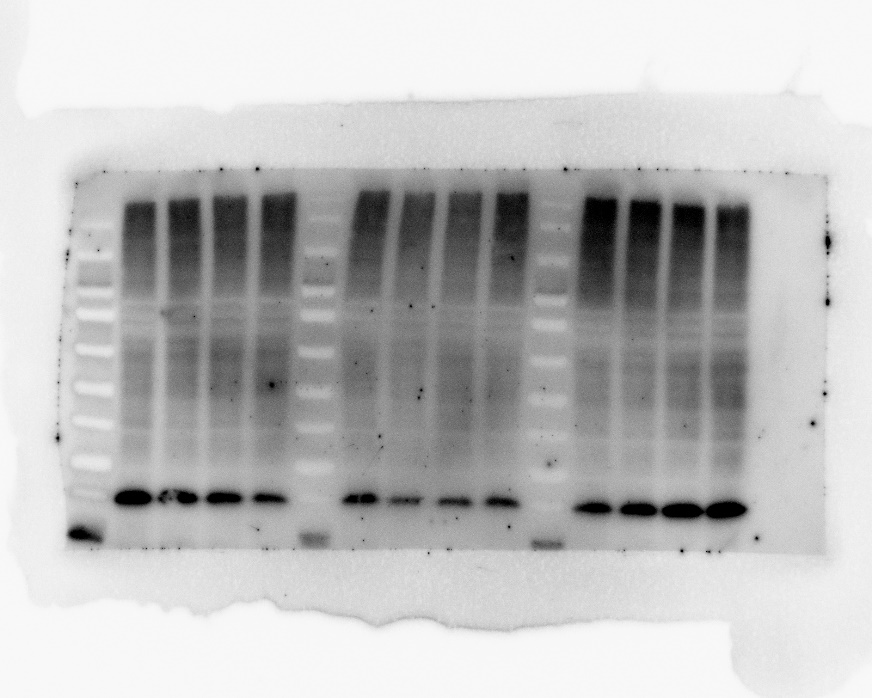


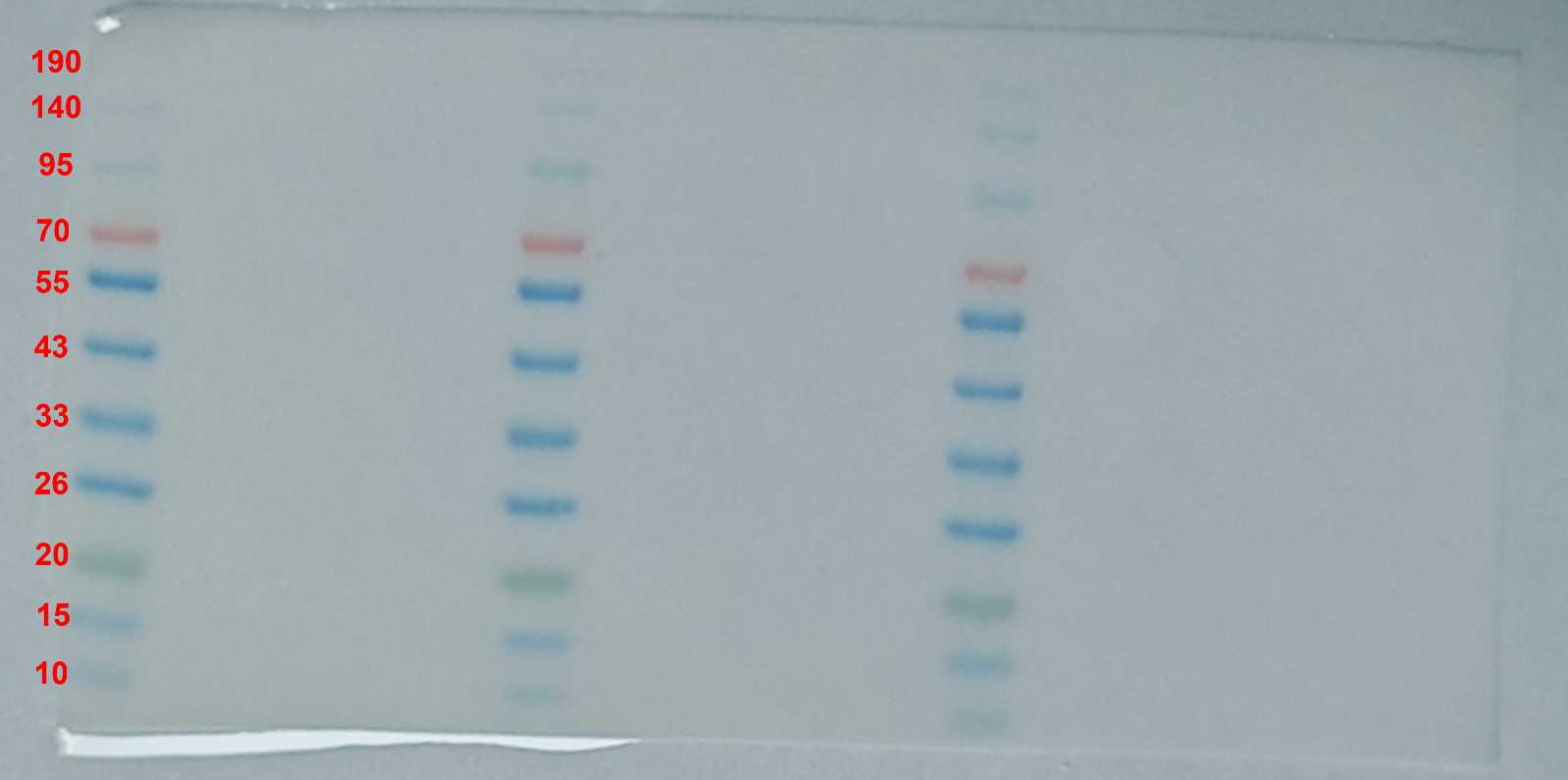

Supplement: S2 File — (DOCX) [file pone.0321121.s002.docx]
